# In silico and in vitro prediction of new synthesized N-heterocyclic compounds as anti-SARS-CoV-2

**DOI:** 10.1038/s41598-024-51443-7

**Published:** 2024-01-11

**Authors:** Heba E. Hashem, Sajjad Ahmad, Ajoy Kumer, Youness El Bakri

**Affiliations:** 1 Department of Chemistry, Faculty of Women, Ain Shams University, HeliopolisCairo, 11757 Egypt; 2 Department of Health and Biological Sciences, Abasyn University, Peshawar, 25000 Pakistan; 3 Department of Natural Sciences, Lebanese American University, P.O. Box 36, Beirut, Lebanon; 4 Gilbert and Rose-Marie Chagoury School of Medicine, Lebanese American University, P.O. Box 36, Beirut, Lebanon; 5 Department of Chemistry, College of Arts and Sciences, IUBAT-International University of Business Agriculture and Technology, Dhaka, 1230 Bangladesh; 6 Center for Global Health Research, Saveetha Institute of Medical and Technical Sciences in Saveetha Medical College and Hospital, Chennai, India; 7 Department of Theoretical and Applied Chemistry, South Ural State University, Lenin Prospect 76, Chelyabinsk, 454080 Russian Federation

**Keywords:** Organic chemistry, Drug discovery, Molecular biology

## Abstract

Computer**-**aided drug design has been employed to get the medicinal effects against Corona virus from different pyridine derivatives after synthesizing the new compounds. Additionally, various computational studies are also employed between the newly prepared pyridine derivatives and three controls against three proteins (6Y2E, 6M71 and 6M3M). Different methods were employed to synthesize new pyridine derivatives according to the literature using different reaction mediums. MTT was performed for cytotoxicity study and IC_50_ for inhibitory concentration. Additionally, in-silico studies including DFT, molecular docking, molecular dynamics, MMPBSA, ADME, pharmacokinetics and Lipinski rules were evaluated. The chemical structures of all new compounds were elucidated based on spectroscopic investigation. A molecular docking study demonstrated that compounds **5**, **11**, and **12** have the best binders of the SARS-CoV-2 main protease enzyme, with energy scores of − 7.5 kcal/mol, − 7.2 kcal/mol, and − 7.9 kcal/mol, respectively. The net binding energy values of the 11-Mpro, 12-Mpro, and 5-Mpro complexes revealed their highly stable nature in terms of both intermolecular interactions and docked conformation across the simulation time. ADME properties, besides the pharmacokinetics and Lipinski rules, showed that all seven newly synthesized compounds follow Lipinski rules with high GI absorption. The In Vitro antiviral study against SARS-CoV-2 using MTT methods confirms that compound **5** has more potential and is safer than other tested compounds. The study shows that the newly synthesized pyridine derivatives have medicinal properties against SARS-CoV-2 without violating Lipinski rules. Compounds **5**, **11,** and **12**, particularly compound **5,** may serve as promising potential candidate for COVID-19.

## Introduction

The SARS-CoV-2 coronavirus has caused a global pandemic, resulting in significant morbidity and mortality worldwide. Scientists and researchers have been motivated to investigate and develop potential treatments due to the severity of the illness and the virus's rapid mutation^1,2^. Developing an effective vaccine and discovering a treatment for the illness have been the primary focuses of research in this field.

Traditionally, chemical medications with synthetic organic chemical bases have been preferred for treating viral infections^3^. However, there has been some criticism of their widespread use in recent years due to their severe side effects.

Medicinal chemists and researchers have been increasingly interested in heterocyclic compounds with five and six-membered rings, including nitrogen, sulfur, and oxygen. These compounds exhibit a variety of biological and pharmacological actions, making them a promising area of study. Pyridine, 1,3,4-thiadiazoles, pyran, and 1,2,4-triazoles are among the most advantageous ring systems for medicinal purposes.

**Figure 1:**
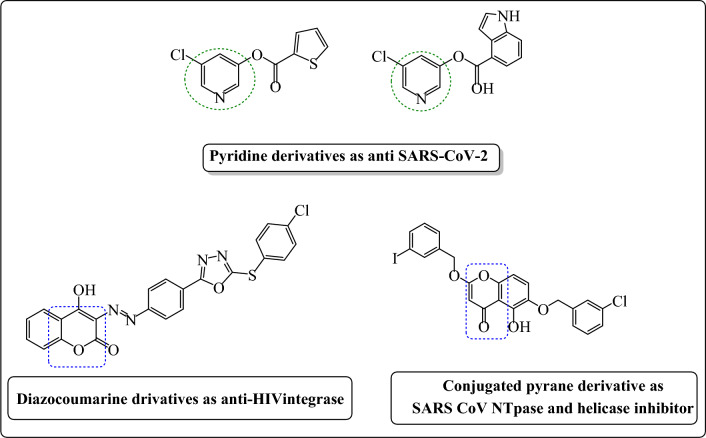
Recently reported pyridine and conjugated pyran based heterocyclic compounds with antiviral and anti-coronavirus activities.

The Pyridine scaffold participates in various compounds producing a wide range of biological activities such as antiviral, antibacterial, antitubercular, and antihistaminic effect^4,5^. Moreover, conjugated pyran play a vital role in drug discovery according to their multiple pharmacological actions, such as antimicrobial^6^, anti-tumor^7^, influenza inhibition^8^ and antiviral^9^ (Fig. 1).

**Figure 2:**
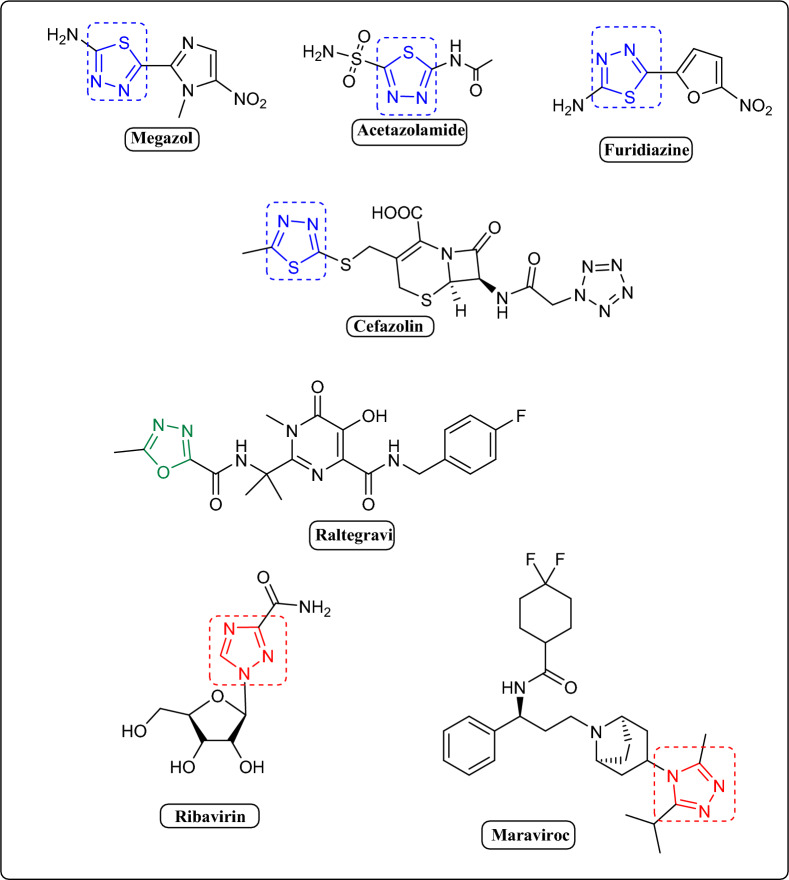
Miscellaneous Marketed antiviral drugs bearing Thiazole, oxadiazole, and triazole moieties.

On the other hand, Nitrogenous five-membered heterocycles have been used in a diverse collection of medications, including antidiabetic^10,11^, antibacterial^12^, anticancer^13,14^, anti-inflammatory^15^, antileishmanial^16^, and antiviral drugs^17^. 1,3,4-thiadiazoles have also been discovered in a number of widely used pharmaceuticals, such as the antiparasitic Megazol, the sodium diuretics acetazolamide, methazolamide, Furidiazine, and the antibiotic Cefazolin^18^. The triazole moiety can be found in a variety of medications, including the antiviral ribavirin and Maraviroc^19^. Currently, oxadiazole derivatives show important biological activities in different area especially as antiviral and antioxidant properties^20–22^. Notwithstanding, Numerous antiviral drugs bearing the 1,3,4-oxadiazole moiety are commercially available such as Raltegravi^23^ (Fig. 2).

Even though there are a limit amount of approved effective inhibitors against SARS-CoV-2, and the full treatment procedure depends on vaccination has some limitations as well. In addition, Viruses, including SARS-CoV-2, can mutate over time, New variants may arise, impacting the efficacy of existing drugs or vaccines. Keeping up with the virus's evolution and ensuring drugs remain effective against these variants pose a significant challenge. Additionally, drug development requires substantial financial investment for research, clinical trials, manufacturing, and distribution. Nonetheless, some agencies -FDA (in the United States) or the EMA (in Europe)—have strict guidelines to ensure the safety, quality, and effectiveness of any drug, which can lead to a lengthy approval process and difficult to make a drug sending available in market. Despite these challenges, numerous treatments and drugs have been developed and repurposed to manage COVID-19, such as remdesivir, dexamethasone, monoclonal antibodies, and vaccines. Researchers and pharmaceutical companies continue to work on developing more effective therapeutics to combat the virus and its variants. The process is ongoing, and scientific advancements are constantly being made in the quest for effective treatments against SARS-CoV-2.

Previous studies have shown that one of the main pharmacophoric features shared by multiple SARS-CoV-2 proteins is pyridine moiety which can be fitted well inside the pocket of protein through H-bond formation with different amino acids. Additionally, an electrophilic centers like amide or imine can participate by forming covalent bonds with different amino acid residues present in the linker region. On the other hand, different five heterocyclic ring such as thiadiazole, tiazole, oxadiazole, and indole have been explored for activity against corona viruses and revealed their high efficiency against different kind of SARS-CoV-2 proteins^24^.

Based on this view, and as an aspect of our continuing research into the discovery and synthesis of novel bioactive heterocyclic compounds^25–34^, the presented work deals with the design and synthesis of new pyridines bearing various heterocycles, as well as the investigation of their pharmacological activity against SARS-CoV-2 through both in vitro and in silico studies.

Computational chemistry techniques are among the most important tools used in drug development today. Compared to wet processes, computational methods require less labor, money, and time. Molecular docking, molecular dynamics simulation, and binding free energy approaches are the most often utilized computational chemistry techniques in this work to predict the binding strength of the synthesized compounds against various SARS-CoV-2 enzymes.

Additionally, by DFT methods, we performed the molecular optimization of the compounds. The motivation for performing a structure optimization was to determine the ground state parameters of the studied system by calculating the optimized parameters (arrangement of the atoms, chemical bond lengths, angles and optimized structure). The calculated parameters can be used in a variety of experimental and theoretical investigations including vibrations, charge distribution, HOMO and LUMO orbital energies, and many more. Further, drug-likeness of the compounds were determined by means of Lipinski’s rule of five so that they are suitable drugs to be marketed.

## Discussion

### Chemistry

**Figure 3:**
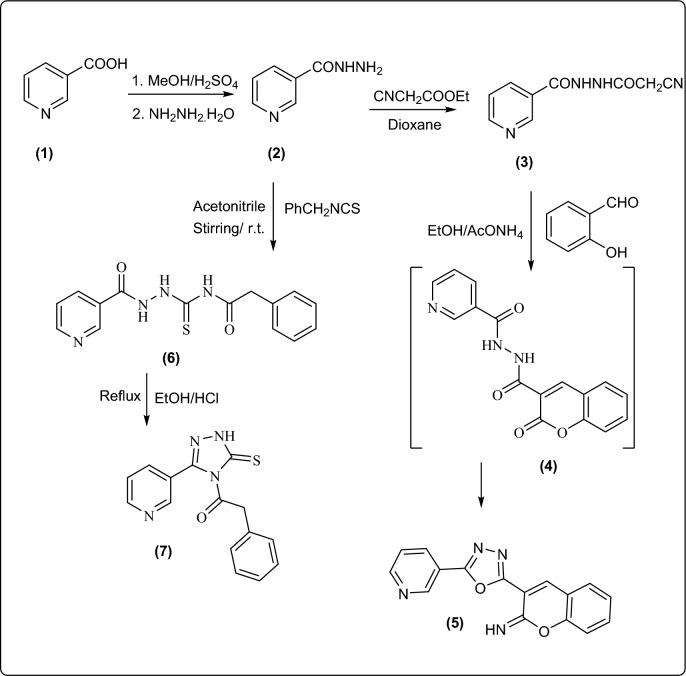
Synthesis of new pyridine attached oxadiazole and triazole rings.

The Key starting active compound, nicotinic acid hydrazide **2**, was synthesized according to the literature method^5^. Equimolar reaction of compound **2** with ethyl cyanoacetate gave the corresponding cyanoacetohydrazide derivative **3**^35^, which cyclized to the corresponding oxadizole-pyran derivative **5** by refluxing with salicaldehyde in ethanol and catalytic amount of ammonium acetate (Fig. 3).

The Infrared spectrum of compound **5** was devoid of any absorption band in characteristic carbonyl group region, instead, it showed an absorption band at 1652 cm^-1^ correlated to the C = N group, which confirmed the cyclization step. Furthermore, proof for the assigned structure of **5** was gained from its ^1^HNMR spectrum which exhibited only one broad singlet signal for NH group besides multiple signals for aromatic protons. Moreover, its ^13^CNMR was in agree with the suggested structure as it exhibited three signals at δ 160.67, 161.21, 163.15 ppm referring to C = N groups (cf. experimental).

On the other hand, synthesis of new pyridine-triazolo-thione derivative **7** was achieved by treating the hydrazide compound **2** with 2-phenylacetyl isothiocyanate at room temperature producing the open adduct thiourea derivative **6**, followed by cyclization in refluxing acidic medium (Fig. 3).

The structure of compounds **6** and **7** were assessed from their spectral data. Their IR spectra represented an additional characteristic band at 1238–1242 cm^−1^ for C = S group. The newly formed triazole ring in compound **7** was established from its ^1^HNMR spectrum which exhibited only one broad absorption band in the downfield region correlated to NH proton. While it represented extra exchangeable broad absorption signals for three NH protons in compound **6**. ^13^C NMR spectra of compounds **6** and **7** were in agree with the suggested structure (cf. experimental).

**Figure 4:**
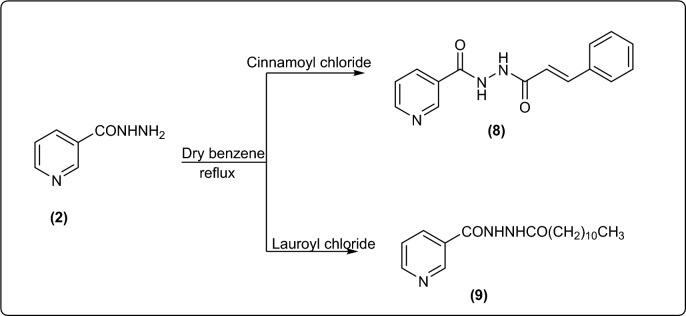
Synthesis of new pyridine carbohydrazide derivatives.

New pyridine carbohydrazide compounds **8** and **9** were synthesized by refluxing equimolar amount of compound **2** with cinnamoyl chloride or lauryl chloride in dry benzene (Fig. 4).

The chemical structures of compounds **8 and 9** were confirmed from their spectral data. Their IR spectra showed characteristic absorption bands for C = O and NH, and CH aliphatic in compound **9.** Moreover, the predicted structures of compounds **8** and **9** were compatible with their ^1^HNMR and ^13^CNMR spectra (cf. experimental).

Further formation of pyridine bearing different heterocycles was achieved through formation of thiadiazol derivative **10** from the reaction of nicotinic acid with thiosemicarbazide in acidic medium^36^.

**Figure 5:**
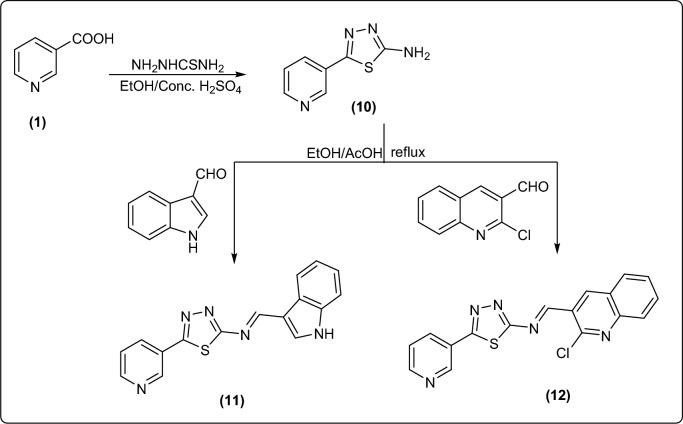
Synthesis of new Schiff base of pyridine-thiadiazol bearing different heterocycles.

Refluxing an equimolar amount of compound **10** with aromatic aldehydes namely indole-3-aldehyde, and 2-chloroquinoline-3-carbaldehyde in ethanol and glacial acetic acid afforded the corresponding Schiff base compounds **11** and** 12**, respectively in an excellent yield (Fig. 5).

IR spectra of compounds **11** and** 12** were devoid of any absorption for amino group, instead, they revealed the presence of bands for C = N groups. Their ^1^HNMR displayed one singlet signals corresponding to azomethine proton N = CH as well as one more broad singlet signal in the downfield region correlated to NH proton in case of compound **11**. (cf. experimental).

### Computational study

#### Molecular optimization and its structure

**Figure 6:**
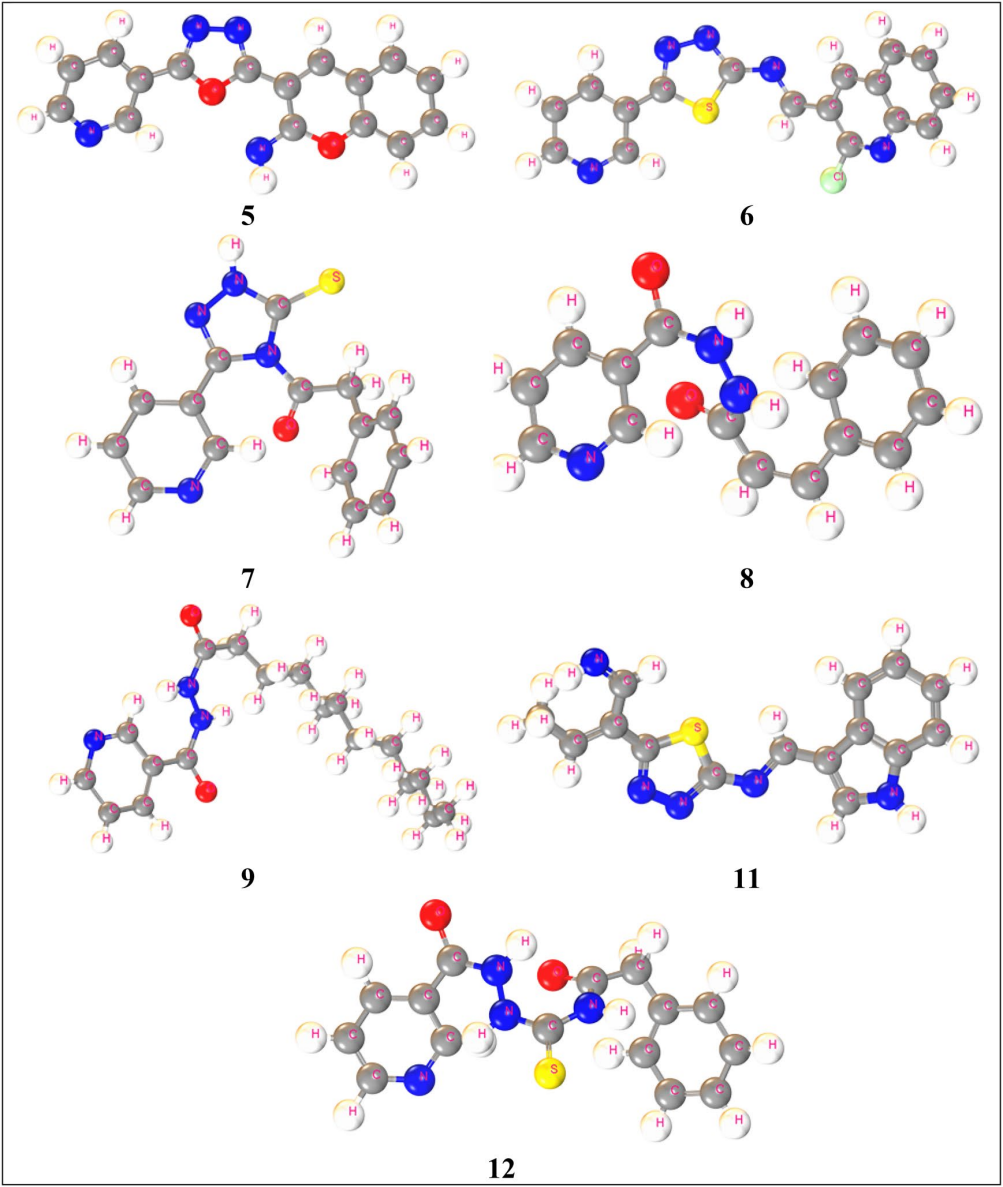
Optimized structure of the new synthesized compounds.

Optimized structure provides the accurate result for the best protein-drug interaction. In this study, the bond length of optimized are as same as standard bond length. The bond length of C(1)-C(5), C(1)-H(23), N(2)-C(3), N(2)-H(22), C(6)-S(7), and C(7)-O(9) after optimization are 1.50 Å, 1.1 Å, 1.5 Å, 1.02 Å, 1.856 Å, and 1.355 Å, which all are almost as same as standard. Optimized structures are depicted in the Fig. 6.

#### HOMO, LUMO, and chemical reactivity descriptors

**Figure 7:**
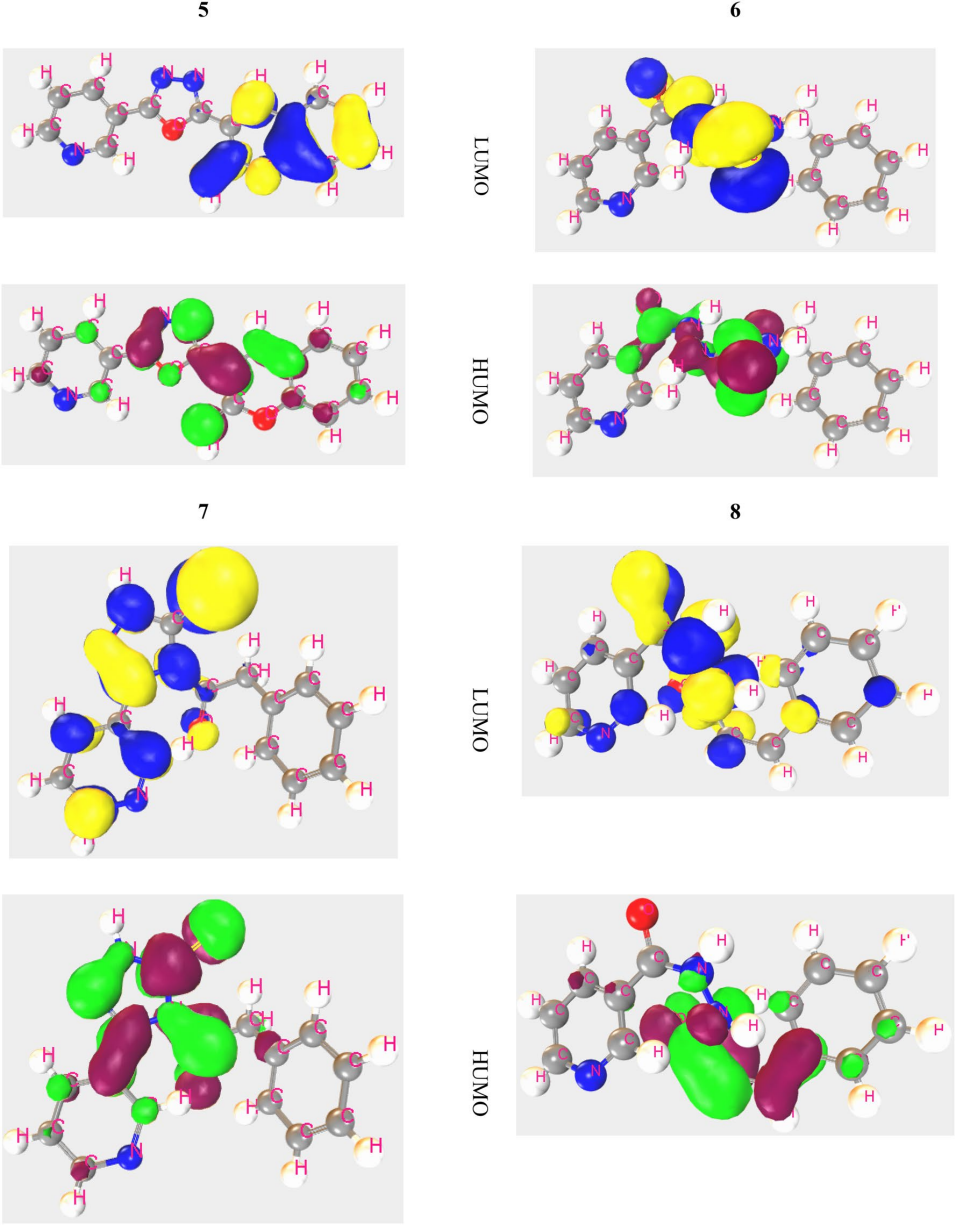

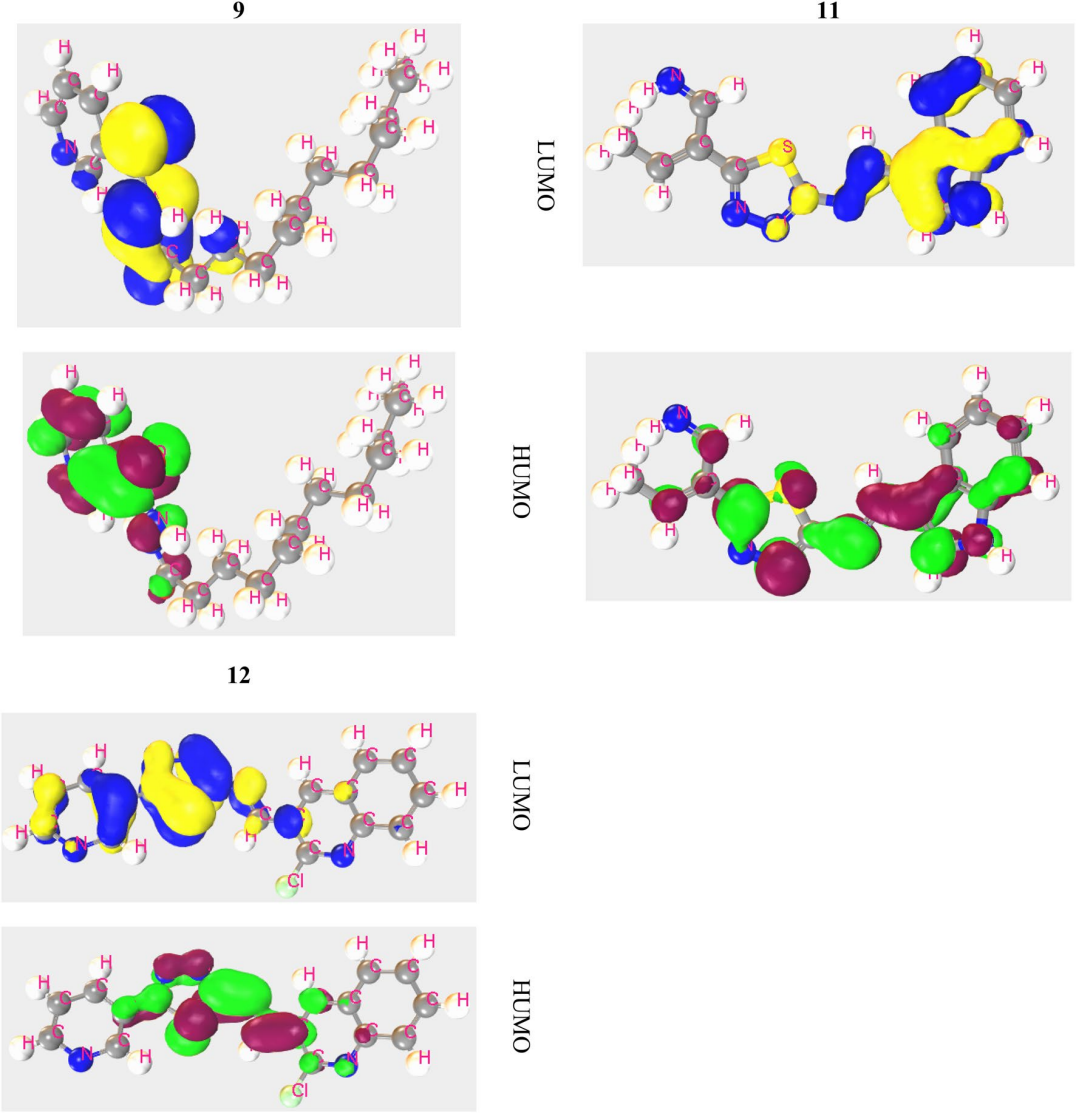
Frontier molecular orbital of HOMO and LUMO.

The particular kind of binding locations and sites of activity where the protein can be banded have been identified by HOMO and LUMO. The positive and negative nodes are symbolized by a variety of hues (Fig. 7). Here, the blue color in HOMO indicates the positive nodes and yellow color donates negative nodes of MFOs. Secondly, Kelly green is positive and dark red is negative. In case of HOMO and LUMO, the orbital spreading area of LUMO is lower region of HOMO. Pursuant to the FMOs diagram, LUMO has been inhabited by functional groups comprising C-N or N–N bonding, whereas HOMO is slightly found in their region. It is capable of functioning as a both electrophilic attracting group material as a result of its raised more LUMO in functional groups. The lower magnitude of energy gap contributes to form an interaction with SARS-CoV-2 protein.

The chemical stability of a molecule has determined by the HOMO–LUMO energy gap. The smaller energy gap, the more stable chemical compound with low dynamics^37^ and functional group is responsible for this Ref.^38^. In the Table 1, the energy gap ranges from − 6.973 to − 7.67 eV with a minimal energy gap. Where energy gap for all compounds is around − 7.0 eV, which represents a lower energy gap as a result, the compounds can be considered as more stable.

**Table 1:** Frontier molecular orbitals and Reactivity descriptor analysis of the new compounds.

Ligand number	I = -HOMO (eV)	A = -LUMO (eV)	E GAP = A-I (eV)	Chemical potential: (μ) = -(I + A)/2 (eV)	Hardness: (η) = (I-A)/2 (eV)	Softness(σ) = 1/μ (eV^-1^)	Electronegativity: (Χ) = (I + A)/2 (eV)	Electrophilicity (ω) = μ^2^/2η (eV)
5	− 9.048	− 1.378	7.670	5.213	3.835	− 0.261	− 3.543	5.213
6	− 8.722	− 1.685	7.037	5.204	3.519	− 0.284	− 3.848	5.204
7	− 9.217	− 2.080	7.137	5.649	3.569	− 0.280	− 4.470	5.649
8	− 9.043	− 1.632	7.411	5.338	3.706	− 0.270	− 3.844	5.338
9	− 9.444	− 1.824	7.620	5.634	3.810	− 0.262	− 4.166	5.634
11	− 8.192	− 1.219	6.973	4.706	3.487	− 0.287	− 3.175	4.706
12	− 9.480	− 2.495	6.985	5.988	3.493	− 0.286	− 5.132	5.988

Better absorption rate depends on hardness and softness, to provide a better absorption rate the value of hardness should be around − 4.00 eV and softness should be lower than hardness. Here, the highest hardness value is − 3.835 eV with the compound **5** and lower value is − 3.487 eV with **11**. This refers that their value is around − 4.00 eV. On the other hand, the value of softness ranges from − 0.261 to − 0.287 eV, which is clearly lower than hardness. According to above data these compounds are stable and have a great absorption rate.

#### Electrostatics potential maps

**Figure 8:**
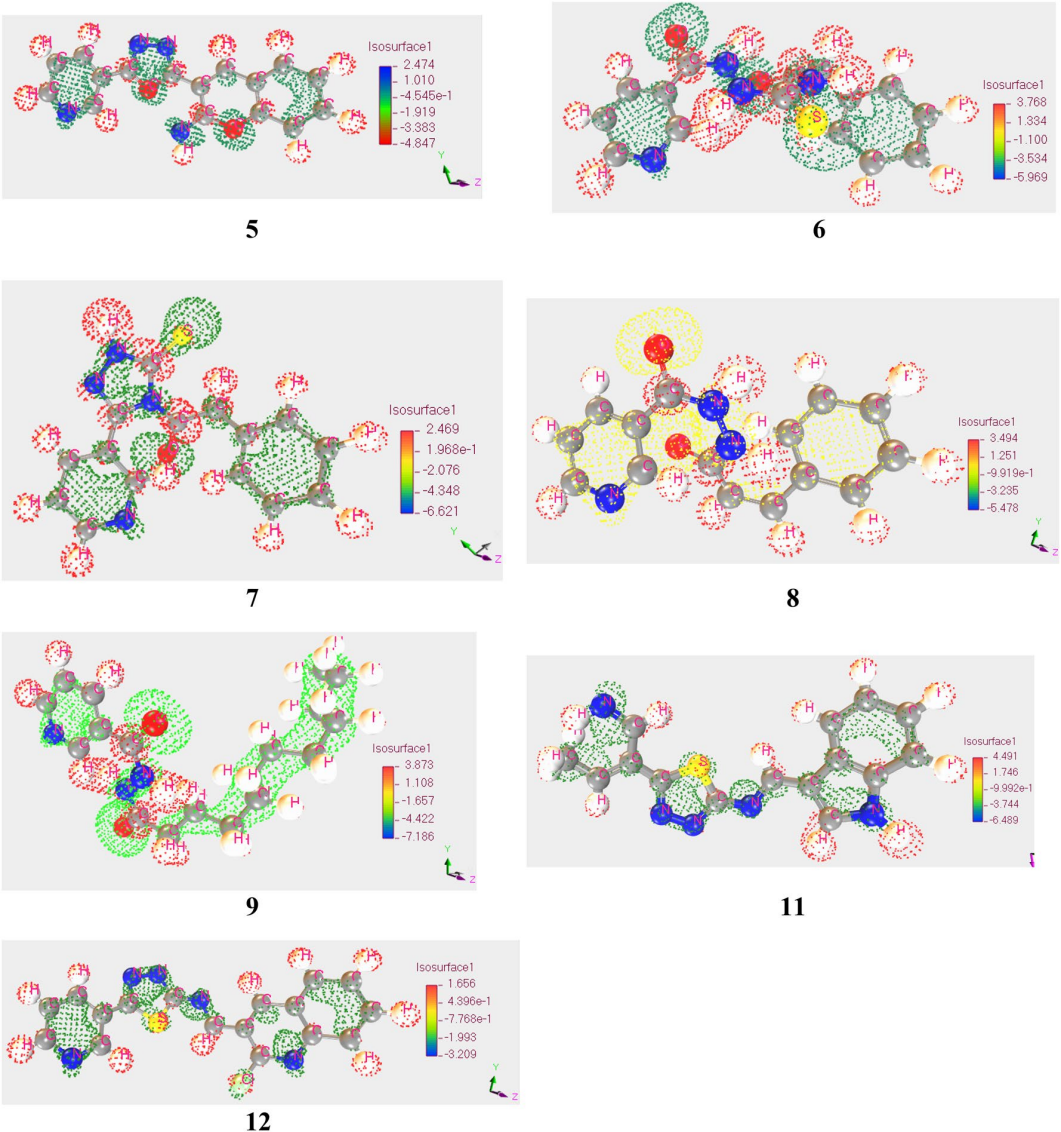
Map of Molecular Electrostatic Potential (MEP) Charge Distribution of reported compounds.

Electrostatics potentiality maps determined the suitable attack side for the compounds whether the compounds will be nucleophilic or electrophilic. The positive and negative sites within the compounds can be determined by this concept^39^. Here, red color demonstrates the positive nodes for **5**, **6**, **7**, **8**, **9**,**11**, and **12** and blue color demonstrates the positive nodes for **5**. On the other hand, blue color describes negative nodes for all the compounds without **5**. Positively charged area is bigger for **5**, **7**, **9**, **12**, and then negatively charged area, while negatively charged area is bigger for **6** and **8** than positively charged area (Fig. 8). As a result, the nucleophilic compound is more desirable for those (**5**, **7**, **11**, **12**, and **9**) compounds whereas electrophilic compounds are desirable for **6** and **8**.

#### Molecular docking

**Figure 9:**
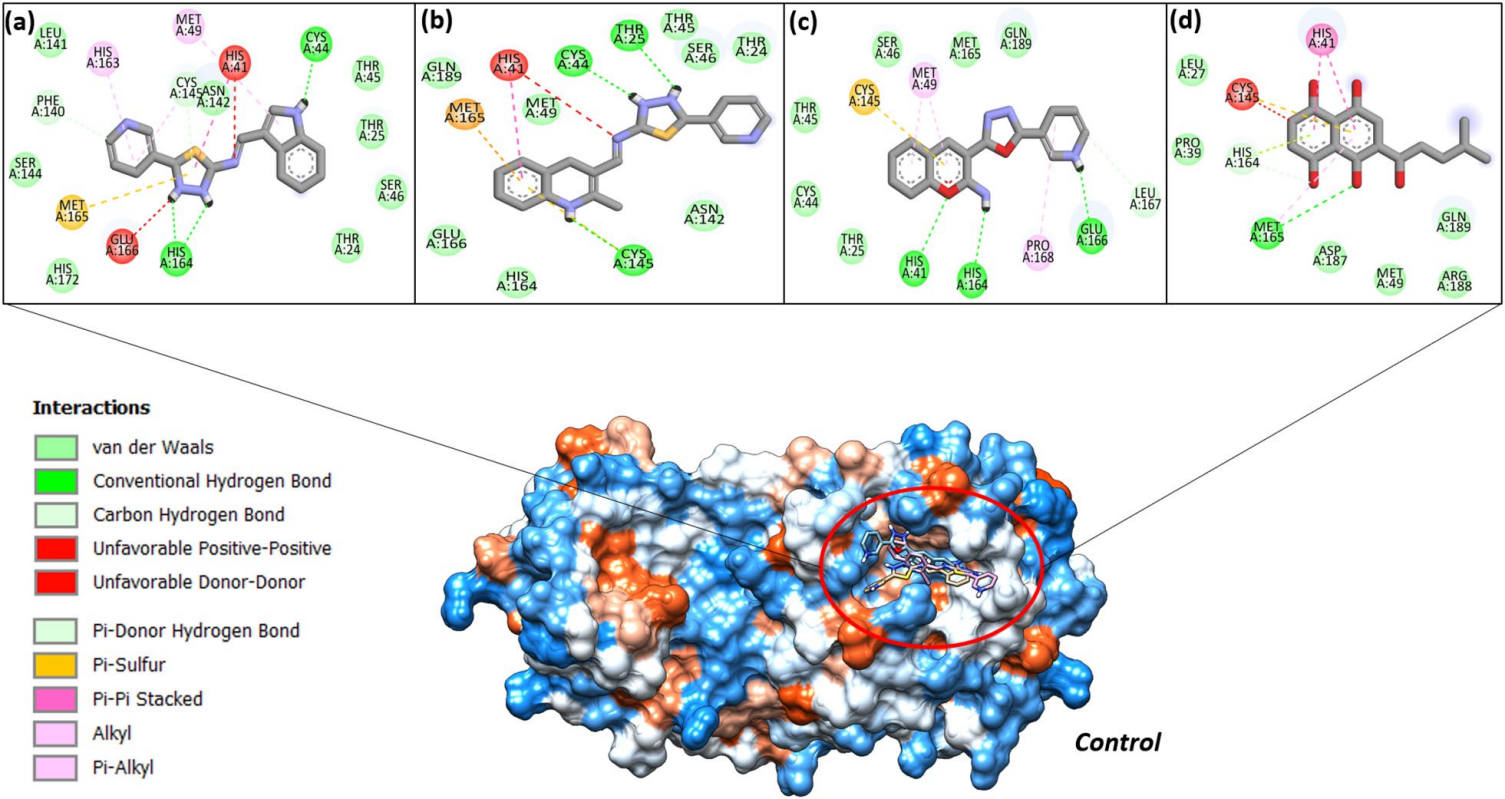
Compounds docked conformation with SARS-CoV-2 main protease enzyme. The enzyme is shown by hydrophobic surface, while compounds are in different colors stick. The **11**, **12** and **5** is in yellow, pink and blue stick, respectively. The interactions of **11** (**a**), **12** (**b**), **5** (**c**) and **control** (**d**) are also given.

Molecular docking is now in routine use application of computer aided drug designing in order to predict intermolecular docking conformation and estimate the binding energy score of docked complexes. The compounds were found to show stable interactions and good affinity for all studies receptors form the SARS-CoV-2 virus. Among the compounds, the **5**, **11** and **12** were prioritized as best binders of SARS-CoV-2 main protease enzyme with energy score of -7.5 kcal/mol, -7.2 kcal/mol and -7.9 kcal/mol, respectively **(**Table 2**)**. All the three compounds were demonstrated to bind the substrate binding pocket of the enzyme with interactions network dominated by both short distance hydrophobic and hydrophilic contacts compared to the studied control (*Alpha-ketoamide inhibitor*) (Fig. 9d). The **11** compound (N-((1H-indol-3-yl)methylene)-5-(pyridin-3-yl)-1,3,4-thiadiazol-2-amine) formed hydrogen bonds with Cys44 and His164 at bond distance of 1.8 Å and 2.1 Å, respectively. Majority of interactions contribution of the compound was observed from 1H-indole and 1,3,4-thiadiazole chemical moiety. The rest of the compound structure produced considerably weak van der Waals bonding. The compound also produce van der Waals contacts with several active site residues as shown in Fig. 9a. The **12** (N-((2-chloroquinolin-3-yl) methylene)-5-(pyridin-3-yl)-1,3,4-thiadiazol-2-amine) was observed to form multiple hydrogen bonds with Thr25, Cys44 and Cys145 at distance length of 1.84 Å, 1.68 Å and 2.1 Å, respectively (Fig. 9b). The central 1,3,4-thiadiazol-2-amine chemical segment of the compound reported to be engaged in major hydrogen bonding compared to the rest of the structure. The compound **5** (3-(5-(pyridin-3-yl)-1,3,4-oxadiazol-2-yl)-2H-chromen-2-imine) formed hydrogen bond with Glu166 (bond distance of 1.1 Å), His164 (bond distance of 2.2 Å), and His41 (bond distance of 2.3 Å) (Fig. 9c). The compound 2*H*-chromen-2-imine and pyridine reported strong hydrogen bonds with the SARS-CoV-2 main protease active site residues. The intermolecular docked conformation and binding interactions of the SARS-CoV-2 RdRP enzyme and Nucleocapsid protein with the compounds **11**, **12** and **5** are provided in S-Figs. 1 and 2, respectively.

**Table 2:** Docking score of compounds with respect to different SARS-CoV-2 receptors in kcal/mol.

Compounds	SARS-Cov-2 main protease enzyme (6Y2E)	Control (Alpha-ketoamide inhibitor)	SARS-CoV-2 RdRP (6M71)	Control (Remdesivir)	Nucleocapsid protein (6M3M)	Control (Folic Acids)
5	− 7.9	− 7.81	− 7.50	− 8.65	− 7.80	− 7.54
6	− 7.2		− 4.39		− 6.38	
7	− 6.8		− 6.33		− 6.39	
8	− 6.3		− 6.00		− 6.34	
9	− 5.8		− 5.39		− 5.88	
11	− 7.5		− 6.32		− 5.98	
12	− 7.0		− 5.87		− 6.84

#### Molecular dynamic simulation

**Figure 10:**
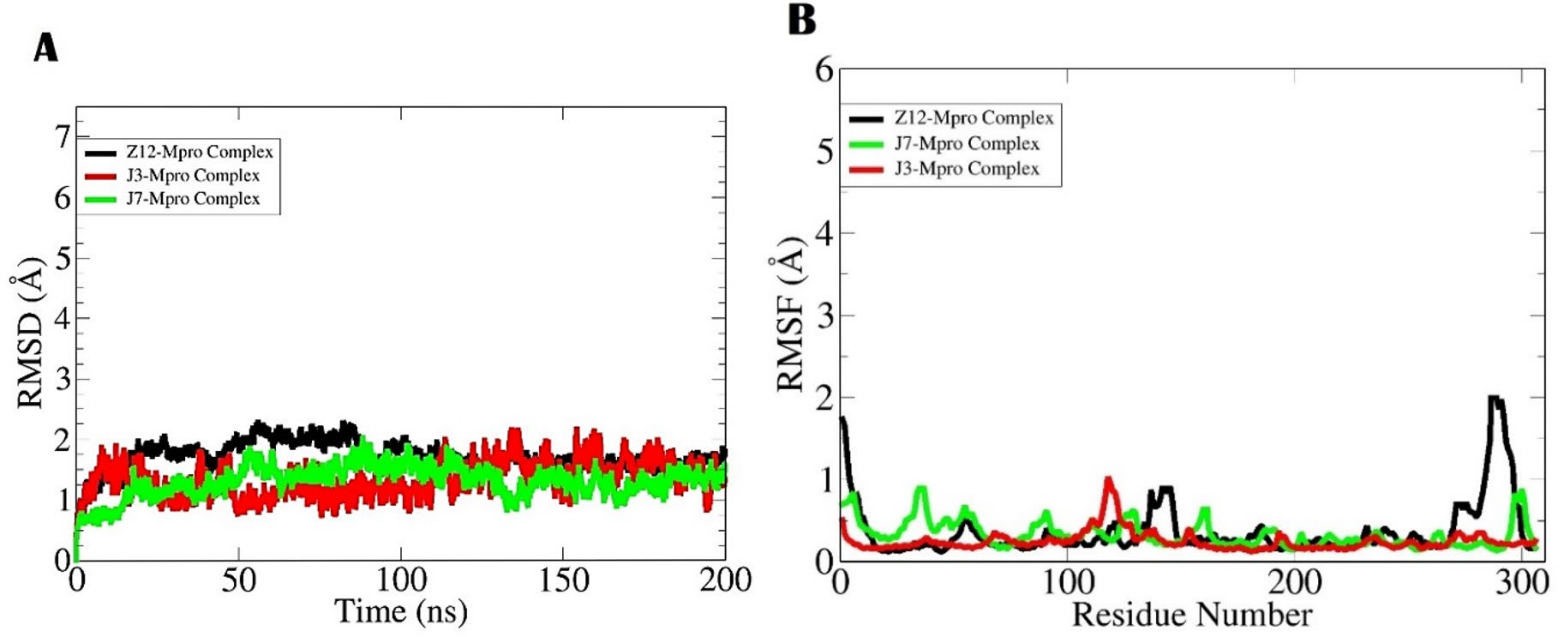
The dynamic simulation analysis of complexes. The first analysis done was RMSD (**A**) and RMSF (**B**). The compounds labeling is done as; **5** = Z12, **11** = J3, and **12** = J7.

The molecular dynamic simulation is a computational based technique to study dynamic properties of biological macromolecule and macromolecule-ligand complexes. The main protease enzyme was seen in considerable stable state in the presence of the compounds as depicted by the root mean square deviation (RMSD) analysis given in Fig. 10A. The mean RMSD of the 11-Mpro complex, 12-Mpro complex and 5-Mpro complex is 1.0 Å, 0.9 Å, and 1.1 Å, respectively. The stable structure of the enzyme illustrates that the compounds binding conformation with the receptor is static and formed multiple strong hydrophilic and hydrophobic contacts. The residue level stability of receptor molecule was understand using root mean square fluctuation (RMSF), which complements the RMSD and demonstrates overall residues level stable behavior of complexes. The 5-Mpro complex showed some smaller N-terminal jumps that were linked to the loops, which are naturally flexible regions. The average RMSF value of 11-Mpro complex, 12-Mpro complex and 5-Mpro complex is 0.7 Å, 0.8 Å and 1.3 Å, respectively (Fig. 10B).

#### Binding free estimation

The binding free energies of top ranked complexes were estimated to validate the intermolecular binding interactions and docked stability. The MM-GBSA method is considered more reliable than the conventional docking studies. The Table 3 tabulates all the binding energy terms estimated for the complexes. As can be observed, the van der Waals energy dominates the overall net energy of complexes. The van der Waals energy of 11-Mpro Complex, 12-Mpro Complex and 5-Mpro Complex is − 45.12 kcal/mol, − 51.37 kcal/mol and − 47.60 kcal/mol, respectively. Likewise, the electrostatic energy term was also found to favor the intermolecular energy. The electrostatic energy of 11-Mpro Complex, 12-Mpro Complex and 5-Mpro Complex is − 16.10 kcal/mol, − 14.0 kcal/mol and − 18.54 kcal/mol, respectively. On the other side, the solvation energy in particular the polar solvation energy is less contributing to overall energy. The net solvation energy for complexes is 7.22 kcal/mol (11-Mpro Complex), 8.08 kcal/mol (12-Mpro Complex) and—3.3 kcal/mol (5-Mpro Complex). The net binding energy value of the 11-Mpro Complex, 12-Mpro Complex and 5-Mpro Complex is − 54 kcal/mol, − 57.29 kcal/mol and − 62.84 kcal/mol, respectively. These values demonstrate the complexes highly stable nature in term of both intermolecular interactions and docked conformation across the simulation time.

**Table 3:** MMGBSA free energy estimation in kcal/mol.

Energy parameter	11-Mpro complex	12-Mpro complex	5-Mpro complex
MM-GBSA			
VDWAALS	− 45.12	− 51.37	− 47.60
EEL	− 16.10	− 14.00	− 18.54
EGB	15.09	16.30	14.54
ESURF	− 7.87	− 8.22	− 11.24
Delta G gas	− 61.22	− 65.37	− 66.14
Delta G solv	7.22	8.08	− 3.3
Delta total	− 54	− 57.29	− 62.84

#### Evaluation of physicochemical and drug likeness properties (Lipinski Rules)

The most acceptable rules for recognizing drug likeness properties are Lipinski rules which is also known as rules of five. Among the five rules the drug candidate should follow at least four rules to be a drug^40^. Commonly acceptable rules are molecular weight, hydrogen bond acceptors and donors, TPSA and log S with the acceptable values are 150 to 500 g/mol, ≤ 10, ≤ 5, 20 to 130 Å^2^ and less than 6, respectively.

The molecular weight for all the compounds is in the referenced value. The highest Molecular weight is 351.81 g/mol and the lowest molecular weight is 267.28 g/mol, for the compounds **12** and **8**, respectively. The hydrogen bond acceptors range from 3 to 6. All the compounds have lower than 10 hydrogen acceptors. **12** has no hydrogen bond donor, rather than that the hydrogen bond donor’s ranges from 1 to 3, which all are acceptable. All the compounds are following the standard TPSA value. **8** and **9** have the lowest value at 71.09 Å^2^ and **6** has the highest value at 115.21 Å^2^. Log S values are in negative which confirms that all are lower than 6 and following the drug likeness properties. The seven-drug candidate is following all the Lipinski rules, there is no violation of Lipinski rules. 0.55 is the standard bioavailability value for a drug^41^. The bioavailability values for the experimented compounds are at 0.55, all the compounds are bioavailable besides that their GI absorption is also high. Table 4 shows that all the experimental compounds are following the drug likeness properties. All of them can be drug candidate in terms of pharmacokinetics and Lipinski rules.

**Table 4:** Data of Lipinski rule, pharmacokinetics and drug likeness of different types of pyridine derivatives.

Ligand number	Molecular weight g/mol	Num. H-bond acceptors	Num. H-bond donors	TPSA (Å^2^)	Log Po/w (Consensus)	Log S (ESOL)	Drug likeness (Lipinski Rules)		Bioavailability	GI absorption
							Follow	Violation		
5	290.28	6	1	88.80	2.59	− 3.53	Yes	0	0.55	High
6	314.36	3	3	115.21	1.57	− 2.92	Yes	0	0.55	High
7	296.35	3	1	95.66	2.44	− 3.23	Yes	0	0.55	High
8	267.28	3	2	71.09	1.65	− 2.63	Yes	0	0.55	High
9	319.44	3	2	71.09	3.65	− 4.11	Yes	0	0.55	High
11	295.36	4	2	106.02	3.04	− 3.48	Yes	0	0.55	High
12	351.81	5	0	92.16	3.93	− 4.88	Yes	0	0.55	High

#### Pharmacokinetics parameters and ADMET properties of pyridine derivatives

ADME is a parameter for a chemical regarding safety as a drug. ADME stands for Absorption, distribution, metabolism, excretion, and Toxicity. The drug candidate should follow the standard of the following: log Kp greater than 0.90 cm/s indicates significant Caco2 permeability. Absorption in the intestines must be greater than 30%, Log Kp greater than − 2.5 indicates minimal skin penetration; for BBB permeability if the log BB value is greater than 0.3, it will readily cross the BBB on the other hand if log BB is less than − 1, it will poorly distributed to the brain, those with a logPS >  − 3 are considered to be unable to enter the CNS^42^.

Only **5** and **12** can cross the Caco2 with the same value at 0.993 cm/s, besides these two compounds no compound can cross the Caco2 because their value is less than 0.90 cm/s., **6**, **7**, **8**, **9** and **11** can cross the Caco2 with the value at, 0.867, 0.866, 0.511, 0.511 and 0.858, respectively. Intestinal absorption rat e is more than 30% for all the compounds. Without **6** and **11** the intestinal absorption rate is more than 90%. **6** and **11** has 76.423and 88.488% intestinal absorption rate, respectively. **5** and **12** has the highest absorption rate at 97.982 and 97.556, respectively. **7**, **8** and **9** has the absorption rate at around 90%. The skin permeability value for all the compound is following the standard. All the compounds have the value higher than − 2.6. The log BB value for BBB permeability is lower than − 1 with the value ranges from − 0.014 to − 0.850, the value refers as poorly distributed to the brain, as a result no compound crosses the BBB. Log PS value for CNS permeability ranges from − 2.003 to − 2.759, which considered as the drug cannot penetrate the CNS. No compounds can inhibit CYP2C9, but all the compounds can inhibit the CYP1A2. No compound can excrete Renal OCT2 substrate (Table 5).

**Table 5:** ADME properties of different types of pyridine derivatives.

Ligand number	Absorption			Distribution		Metabolism		Excretion	
	Caco2 permeability (log Papp in 10–6 cm/s)	Intestinal absorption (% Absorbed)	Skin permeability (log Kp)	BBB permeability (log BB)	CNS permeability (log PS)	CYP2C9 inhibitor	CYP1A2 inhibitor	Total Clearance (log ml/min/kg)	Renal OCT2 substrate
5	0.993	97.982	− 2.652	− 0.760	− 2.158	No	Yes	0.366	No
6	0.867	76.423	− 3.109	− 0.850	− 2.759	No	Yes	− 0.308	No
7	0.866	95.295	− 2.815	0.127	− 2.316	No	Yes	0.085	No
8	0.511	94.655	− 2.843	− 0.037	− 2.445	No	Yes	0.219	No
9	0.511	94.655	− 2.843	− 0.037	− 2.445	No	Yes	0.219	No
11	0.858	88.488	− 2.873	− 0.412	− 2.135	No	Yes	0.331	No
12	0.993	97.556	− 2.685	− 0.014	− 2.003	No	Yes	0.158	No

Toxicity is the harmful effect of drug on living organism^43^. Toxicity of the new synthesized compounds including aquatic and non-aquatic toxicity are listed in Table 6. Only the ligand **7** has the positive AMES toxicity, no other ligands have positive AMES toxicity. **5**, **6**, **7**, **11**, and **12** have to risk of hepatic impairment because they have negative hepatotoxicity values, but **9** is risky for hepatic impairment. No compounds have skin sensation, they are safe for skin. Maximum tolerated dose for all the compound without **9** is very low. Oral rat acute toxicity, Oral Rat Chronic Toxicity, and T. Pyriformis toxicity are negative for all the compounds. At the end, only one compounds has AMES toxicity (**7**) and another one compound has Hepatotoxicity (**9**). Otherwise, rest of the compounds have no adverse effect. **5**, **6, 11, and 12** have no toxicity, they are safe for the animal health.

**Table 6:** Aquatic and non aquatic toxicity of the new synthesized compounds.

ligands	AMES toxicity	Hepato- toxicity	Skin sensation	Maximum tolerated dose	Oral rat acute toxicity (LD50)	Oral rat chronic Toxicity (LOAEL)	T. Pyriformis toxicity (Log mg/L)
5	No	No	No	− 0.229	2.153	0.649	0.29
6	No	No	No	0.130	2.157	1.643	0.576
7	Yes	No	No	− 0.135	2.527	1.428	0.436
9	No	Yes	No	0.559	2.255	1.30	1.048
11	No	No	No	0.174	2.586	0.965	0.808
12	No	No	No	− 0.173	2.216	0.698	0.294

### In vitro antiviral anti-COVID-19 biological activity

Based on the results obtained from molecular docking and molecular dynamic studies. among the seven tested compounds, the three compounds **5**, **11**, and **12** with the highest binding energy score were investigated for the term of cytotoxicity concentration 50 (CC50) and Inhibitory concentration 50(IC_50_) against SARS-CoV-2.

It is well known concept of literature for 50% cytotoxicity concentrations (CC50) carried on the concentration of test compounds necessitated to reduce cell viability by 50%. Accurately, cytotoxicity of the test compounds is unsurpassed governed simultaneously with clean cells to obtain the 50% of CC50 values. However, it can be defined as the concentration of tested sample where 50 percent of the host cells were killed by the antiviral product. We strongly acclaim for determining the 50% of CC50 values in both stationary and dividing cells from multiple germane human cell types and tissues to establish the potential for cell-cycle, species, or tissue-specific toxicities. On the other hand, in-vitro susceptibility of viruses to any types ofantiviral agents is stereotypically and emperically measured as the inhibitory concentration 50% (IC_50_), So it is may be defined as the concentration for growth for lowers 50% of the virus-induced cytopathic effect (CPE) and the number of plaques formed.

**Figure 11:**
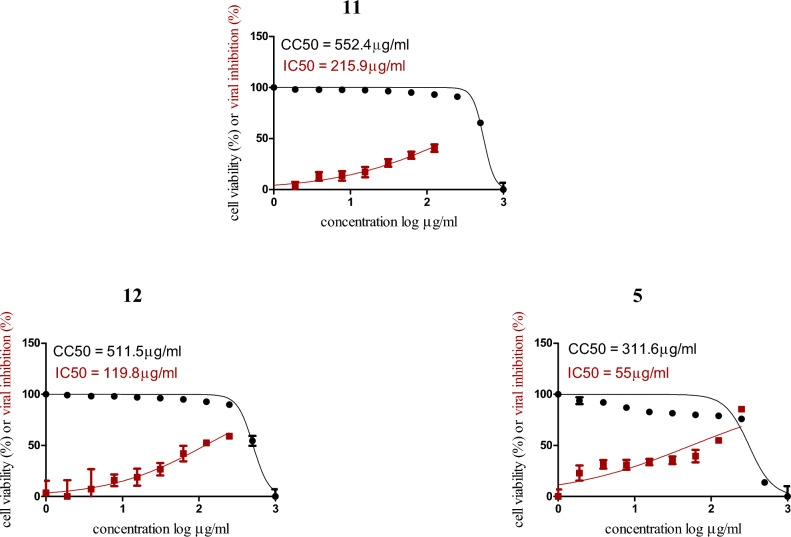
Graph of Cytotoxicity concentration 50 (CC50) and Inhibitory concentration 50(IC_50_) for the new synthesized compounds as anti-SARS-CoV-2.

The cytotoxic activity of the three compounds were tested in VERO-E6 cells by using the 3-(4, 5-dimethylthiazol -2-yl)-2, 5-diphenyltetrazolium bromide (MTT) method^44^. The result of the in vitro anti-SARS-CoV-2 activity revealed that compound **5** has the highest antiviral activity against (hCoV-19/Egypt/NRC-03/2020) at the highst safe tested concentration and decreased gradually (Fig. 11).

## Experimental

### Chemistry

The instruments used for measuring the melting points, spectral data (IR, mass, ^1^HNMR and ^13^CNMR) and elemental analysis are provided in detail in the Supplementary Information.

*Synthesis of nicotinic acid hydrazide derivatives (2) and (3)* were performed according to the reported methods in the literature^5,35^

#### Synthesis of 3-(5-(pyridin-3-yl)-1,3,4-oxadiazol-2-yl)-2H-chromen-2-imine (5)

A mixture of cyanoacetohydrazide derivative **3** (0.01 mol) and salicylaldehyde (0.01 mol) was refluxed in boiling ethanol (25 ml) and in presence of catalytic amount of ammonium acetate for 4 h. The reaction mixture cooled at room temperature and poured gradually onto ice/water. The solid formed was filtered off and recrystallized from ethanol to give compound **5** (Yield 65%); reddish brown crystals; m.p. 199 -200 °C; IR (KBr) (υ, cm^-1^): 3207 (NH), 3093 (CH-Ar), 1652 (C = N); ^1^H NMR (DMSO-d6) δ: 6.98–7.58 (m, 4H, ArH, J = 7.28, 7.63), 8.21 (s, 1H, = CH-Pyran), 7.76–9.09 (m, 4H, = CH-pyridine, J = 7.98, 8.22), 9.35 (br.s, 1H, NH, D_2_O exchangeable); ^13^CNMR (DMSO-d6) δ: 104.11, 112.95, 117.34, 119.99, 124.95, 125.16, 133.74, 134.23, 136.92, 147.20, 152.30, 154.79, 157.24, 160.67, 161.21, 163.15; MS (70 eV) m/z (%): 290 (M^+^, 31). Anal. calcd. for C_16_H_10_N_4_O_2_ (290.28): C, 66.20; H, 3.47; N, 19.30. Found: C, 66.12; H, 3.37; N, 19.25.

#### Synthesis of N-(2-nicotinoylhydrazine-1-carbonothioyl)-2-phenylacetamide (6)

A mixture of 2-phenylacetyl isothiocyanate (0.01 mol) and nicotinic acid hydrazide **2** (0.01 mol) was stirred in acetonitrile (30 ml) at room temperature for 2 h, then heated under reflux for 2h. The reaction mixture cooled at room temperature and poured onto ice water and left overnight. The solid formed was filtered off and recrystallized from ethanol to give compound **6** (Yield 75%); pale yellow crystals; m.p. 180–182 °C; IR (KBr) (υ, cm^−1^): 3158 (NH), 3009 (CH-Ar), 2921, 2849 (CH-aliphatic), 1685, 1650 (C = O), 1152 (CS); ^1^H NMR (DMSO-d6) δ: 3.81 (s, 2H, CH_2_-Ph), 7.27–7.55 (m, 5H, Ar–H, J = 7.27), 7.56–9.03 (m, 4H, = CH-pyridine, J = 7.57, 8.31), 11.26 (br.s, 1H, CONHNHCS, D_2_O exchangeable), 11.82 (br.s, 1H, CONHCS, D_2_O exchangeable), 12.02 (br.s, 1H, CONHNHCS, D_2_O exchangeable); ^13^CNMR (DMSO-d6) δ: 42.60, 124.17, 127.50, 128.43, 128.94, 129.85, 134.72, 135.95, 148.96, 153.04, 163, 74, 172.84, 181.27; MS (70 eV) m/z (%): 314 (M^+^, 100). Anal. calcd. for C_15_H_14_N_4_O_2_S (314.36): C, 57.31; H, 4.49; N, 17.82. Found: C, 57.18; H, 4.39; N, 17.74.

#### Synthesis of 2-phenyl-1-(3-(pyridin-3-yl)-5-thioxo-1,5-dihydro-4H-1,2,4-triazol-4-yl)ethan-1-one (7)

Ethanolic solution of compound **6** (0.01 mol) in presence of 25% HCl, was heated under reflux for 3h. The reaction mixture cooled at room temperature and poured onto ice water. The solid formed was filtered off and recrystallized from ethanol to give compound **7** (Yield 75%); off-white crystals; m.p. 160–162 °C; IR (KBr) (υ, cm^−1^): 3260 (NH), 3091 (CH-Ar), 2900 (CH-aliphatic), 1695 (C = O), 1185 (CS); ^1^H NMR (DMSO-d6) δ: 4.24 (s, 2H, CH_2_-Ph), 7.24–7.89 (m, 5H, Ar–H, J = 7.30), 8.58 (dd, 1H, = CH-pyridine, J = 8.68), 8.82 (d, 1H, = CH-pyridine) 8.97 (d, 1H, = CH-pyridine), 9.15 (s, 3H, = CH-pyridine), 11.16 (br.s, 1H, NH, D_2_O exchangeable); ^13^CNMR (DMSO-d6) δ: 41.21, 127.03, 129.35, 129.77, 135.39, 136.31, 140.49, 141.90, 144.71, 147.11, 150.73, 151.58, 164.43, 170.49, 173.10; MS (70 eV) m/z (%): 296 (M^+^, 53). Anal. calcd. for C_15_H_12_N_4_O_S_ (296.07): C, 60.80; H, 4.08; N, 18.81. Found: C, 59.81; H, 4.12; N, 18.32.

#### Synthesis of N'-cinnamoylnicotinohydrazide (8) and N'-dodecanoylnicotinohydrazide (9)

A mixture of nicotinic acid hydrazide **2** (0.01 mol) and cinnamoyl chloride (0.01 mol) or lauroyl chloride (0.01 mol) was heated under reflux in dry benzene (20 mol) for 2h. The reaction mixture cooled at room temperature, the solid product formed was filtered off and recrystallized from benzene to give the corresponding hydrazide derivatives **8** and **9**, respectively.

*N'-cinnamoylnicotinohydrazide 8* (Yield 82%); brown crystals; m.p. 238–240 °C; IR (KBr) (υ, cm°^1^): 3185, 3118 (NH), 3056, 3022 (CH-Ar), 1673, 1633 (C = O); ^1^H NMR (DMSO-d6) δ: 6.52 (d, 1H, = CH, J = 6.54), 6.81 (d, 1H, = CH, J = 6.81), 6.44–7.65 (m, 6H, Ar–H +  = CH-pyridine, J = 7.76), 8.63 (d, 1H, = CH-pyridine, J = 8.62), 8.94 (d, 1H, = CH-pyridine, J = 8.95), 9.23 (s, 1H, = CH-pyridine), 10.48 (br.s, 1H, NH, D_2_O exchangeable), 11.07(br.s, 1H, NH, D_2_O exchangeable) ; ^13^CNMR (DMSO-d6) δ:119.52, 126.54, 128.27, 129.21, 129.40, 129.49, 130.48, 130.81, 134.85, 141.23, 141.63, 144.42, 147.74, 162.62, 164.87; MS (70 eV) m/z (%): 267 (M^+^, 35). Anal. calcd. for C_15_H_13_N_3_O_2_ (267.10): C, 67.40; H, 4.90; N, 15.72. Found: C, 67.26; H, 4.84; N, 15.48.

*N'-dodecanoylnicotinohydrazide* **9** (Yield 96%); white crystals; m.p. 128–130 °C; IR (KBr) (υ, cm^-1^): 3211 (NH), 3037 (CH-Ar), 2955, 2918, 2848 (CH-aliphatic), 1667 (C = O); ^1^H NMR (DMSO-d6) δ: 0.87 (t, 3H, CH_3_), 1.25 (m, 16H, 8CH_2_), 1.59 (m, 2H, CH_2_), 2.19 (t, 2H, CH_2_CO), 7.66–9.08 (m, 4H, = CH-pyridine, J = 7.68, 8.37), 9.96 (br.s, 1H, NHCOCH_2_, D_2_O exchangeable), 10.60 (br.s, 1H, NHCOAr, D_2_O exchangeable); ^13^CNMR (DMSO-d6) δ: 14.38, 22.55, 25.51, 29.43, 31.75, 33.69, 125.12, 129.47, 146.93, 150.65, 163.74, 172.16; MS (70 eV) m/z (%): 319 (M^+^, 20). Anal. calcd. for C_18_H_29_N_3_O_2_ (319.23): C, 67.68; H, 9.15; N, 13.15. Found: C, 67.34; H, 8.96; N, 13.56.

#### 5-(pyridin-3-yl)-1,3,4-thiadiazol-2-amine (10) was synthesized according to the reported method in the literature^36^

##### Synthesis of 1-(1H-indol-3-yl)-N-(5-(pyridin-3-yl)-1,3,4-thiadiazol-2-yl)methanimine (11) and 1-(2-chloroquinolin-3-yl)-N-(5-(pyridin-3-yl)-1,3,4-thiadiazol-2-yl)methanimine (12)

An equimolar amount of compound **10** and the heterocyclic aldehydes indole-3-aldehyde or 2-chloro-quinoline-3-aldehyde in absolute ethanol (20 ml) and drops of acetic acid glacial, were heated under reflux for 3-4h. The solid products formed while cooling at room temperature were filtered off and recrystallized from ethanol to give **11** and **12**, respectively.

*1-(1H-indol-3-yl)-N-(5-(pyridin-3-yl)-1,3,4-thiadiazol-2-yl)methanimine* **11** (Yield 85%); white crystals; m.p. 240–250 °C; IR (KBr) (υ, cm^−1^): 3210 (NH), 3007 (CH-Ar), 1670 (C = N); ^1^H NMR (DMSO-d6) δ: 7.57–8.31 (m, 5H, ArH-indole ring +  = CH-pyridine) 7.61 (s, 1H, NCH = , indole), 8.71 (d, 1H, = CH-pyridine), 8.80 (d, 1H, = CH-pyridine), 9.10 (s, 1H, = CH-pyridine), 9.27 (s, 1H, CH = N); 10.88 (br.s, 1H, NH, D_2_O exchangeable); ^13^CNMR (DMSO-d6) δ: 165.11, 153.02, 149.70, 148.78, 147.70, 136.70, 135.89, 134.96, 128.54, 124.93, 124.33, 124.21; MS (70 eV) m/z (%): 305 (M^+^, 52). Anal. calcd. for C_16_H_11_N_5_S (305.36): C, 62.93; H, 3.63; N, 22.94. Found: C, 62.86; H, 3.59; N, 22.84.

*1-(2-chloroquinolin-3-yl)-N-(5-(pyridin-3-yl)-1,3,4-thiadiazol-2-yl)methanimine* **12** (Yield 93%); pale brown crystals; m.p. 288–290 °C; IR (KBr) (υ, cm^−1^): 3005 (CH-Ar), 1673 (C = N), 1634 (C = C); ^1^H NMR (DMSO-d6) δ: 7.22–89 (m, 4H, ArH-quinoline ring) 8.29–8.78 (m, 4H, CH-pyridine), 9.11 (s, 1H, CHquinoline), 10.23 (s, 1H, CH = N); ^13^CNMR (DMSO-d6) δ: 115.94, 123.15, 124.20, 124.82, 125.93, 128.18, 128.55, 130.61, 131.29, 134.85, 135.81, 136.64, 141.57, 143.12, 148.93, 153.02, 161.86, 164.97; MS (70 eV) m/z (%): 351 (M^+^, 44). Anal. calcd. for C_17_H_10_ClN_5_S (351.81): C, 58.04; H, 2.87; N, 19.91. Found: C, 58.12; H, 2.79; N, 19.85.

### Molecular optimization and evaluation of FMOs, and chemical descriptors

Structure optimization and DFT was permed by material studio version 8.0 with the DMol3 and hybrid functional of B3LYP^45^.

The main rationale of the Becke, 3-parameter Lee–Yang–Parr (B3LYP) method is widely used for DFT calculations because it is highly capable of giving the accurate results for the electronic structure and quantum calculations. The precise estimation of electronic properties, as well as the electron transport potential by evaluating the electrostatic 3D map, was obtained using DFT in this study. The HOMO (highest occupied molecular orbital) and LUMO (lowest unoccupied molecular orbital) are two frontier molecular orbitals that have been used to predict chemical descriptors, such as electrophilicity (ω), chemical potential (μ), electronegativity (**Χ**), hardness (**η**), and softness (S). The ionization energy (Ι) and electron affinity (A) can be determined using I = –HOMO and A = –LUMO values, respectively. More details were provided in Supplementary Information.

### Molecular docking

The docking study was initiated by retrieving crystal structure of SARS-CoV-2 main protease enzyme (6Y2E), RdRP (6M71) and nucleocapsid protein (6M3M) from protein data bank^46^. The structures were processed in UCSF Chimera v1.16 by subjecting to energy minimization process^47^. Briefly, missing hydrogen atoms were added first and then charge assignment was done using Gasteiger method. The energy minimization was conducted using steepest descent and conjugate gradient methods for total of 1000 steps. On the other side, the compounds were drawn in ChemDraw Ultra 12.0 and then energy minimized using MM2 force field in Chem3D pro 12.0^48^. Molecular docking was performed in PyRx 0.8 using AutoDock 4.0 software^49,50^. The active site information of main protease enzyme, RDRP and nucleocapsid protein was collected from published literature^51,52^. The grid box dimensions set around the active box was set 25 Å along the XYZ axis. The active site residue coordinates used in docking for the main protease enzyme, RdRP and nucleocapsid protein was His41, Asn496 and Phe286, respectively. The number of docked poses generated for each compound was set to 100. The best docked complexes were measured in term of binding energy in kcal/mol. The docked complexes were visualized via Discovery Studio v 2021^53^ and UCSF Chimera v 1.16^47^.

### Molecular dynamic simulation and MMPBSA analysis

Molecular dynamic simulation was performed for the three top complexes using AMBER20 simulation software^54^. The simulation protocol was accomplished using steps described by Ahmad et al., 2017^55^. The initial processing of the complexes was done through Antechamber program of AMBER20^56^. The receptor protein was prepared using FF14Sb force field while the compounds were prepared via GAFF force field^57^. The molecular dynamics simulations were conducted in three phases; prmtop files generation, preprocessing and production run. The selected docked complexes were first submerged into TIP3P water box, where counter ions were added to get neutral systems. The complexes were then heated gradually to 310 K, then equilibrated and subjected to a production run of 200 ns. The temperature control was achieved using Langevin dynamics^58^. The hydrogen bonds were constrained by SHAKE algorithm^59^. The CPPTRAJ was employed to investigate structure dynamics of complexes^57^. The XMGRACE v5.1 was considered for plotting simulation graphs^60^. The binding free energy of complexes were estimated using MM-GBSA method and accomplished through MMPBSA.py module of the AMBER software^61,62^. In total, 1000 frames were picked from the simulation trajectories and analyzed.

### Determination of pharmacokinetics, Lipinski rule, and toxicity

The pharmacokinetics properties or ADME properties including Absorption, distribution, metabolism, excretion, Lipinski rules, and toxicities are collected from two different online database named SwissADME (http://www.swissadme.ch/)^40^ and another online tool pkCSM (http://biosig.unimelb.edu.au/pkcsm/)^42^. Those data were collected by using the SMILES and the smiles was generated from SwissADME by using the mol files of the compounds.

### In vitro MTT cytotoxicity assay

To assess the half maximal cytotoxic concentration (CC50), stock solutions of the test compounds were prepared in 10% DMSO in ddH2O and diluted further to the working solutions with DMEM. The cytotoxic activity of the extracts was tested in VERO-E6 cells by using the 3-(4, 5-dimethylthiazol -2-yl)-2, 5-diphenyltetrazolium bromide (MTT) method with minor modifications. More details were provided in Supplementary Information.

### Inhibitory concentration 50 (IC_50_) determination

In 96-well tissue culture plates, 2.4 × 10^4^ Vero-E6 cells were distributed in each well and incubated overnight at a humidified 37 °C incubator under 5%CO2 condition. The cell monolayers were then washed once with 1 × PBS and subjected to virus adsorption (hCoV-19/Egypt/NRC-03/2020 (Accession Number on GSAID: EPI_ISL_430820)) for 1 h at room temperature (RT). More details were provided in Supplementary Information.

### Ethics declarations

All experiments were performed in accordance with relevant named guidelines and regulations. No human participants/ human cells, tissues or animals were involved in the studies.

## Conclusion

Experimental and computational method is used to experiment the medicinal effects of pyridine derivatives against COVID-19. Firstly, Synthesis was performed to get the desired compound by employing different methods. Frontier molecular orbitals and Reactivity descriptor analysis confirms the better stability and absorption rate. Then, molecular docking of seven pyridine derivatives against 6Y2E, 6M71 and 6M3M are performed and get highest docking results at of − 7.5 kcal/mol, − 7.2 kcal/mol and − 7.9 kcal/mol, for **11**, **12** and **5**, respectively. Furthermore, the molecular dynamics was performed, and it verified the protein–ligand binding is stable. MMGBSA was performed to validate the intermolecular binding interactions and docked stability according to RMSD and RMSF value. This experiment confirms that all the top complexes are highly stable in nature at the time of simulation. It also confirms that **11**, **12** and **5** is more stable and active than other. The cytotoxicity and IC_50_ test were performed to check the safety and potentiality of the most potent compounds. MTT test confirms compound **5** is safer than other. After that, ADME properties was collected from web server besides that pharmacokinetics and Lipinski rules are also calculated. All the seven compounds follow Lipinski rules, there is no violation and GI absorption is high. To conclude, considering all the factors, compounds **5**, **11** and **12** can be potential drug for COVID-19, especially compound **5**.

### Supplementary Information

Supplementary Information.

## Data Availability

Data generated or analyzed during this study are included in this published article [and its supplementary information files].
