# Supplementary material for: In silico and in vitro prediction of new synthesized N-heterocyclic compounds as anti-SARS-CoV-2
Source: Sci Rep. 2024 Jan 11;14:1152. doi: 10.1038/s41598-024-51443-7 (PMC10784557; doi:10.1038/s41598-024-51443-7)
Supplement: Supplementary file 1 — Supplementary Information. [file 41598_2024_51443_MOESM1_ESM.pdf]

Supplementary data

In silico and In Vitro Prediction of New Synthesized N-heterocyclic Compounds as Anti-SARS-CoV-2

***Heba E. Hashem*[1]*, Sajjad Ahmad[2.3.4], *Ajoy Kumer* [5,6], Youness El Bakri[7]**

1. Department of Chemistry, Ain Shams University, Faculty of Women, Heliopolis, 11757, Cairo, Egypt.
2. Department of Health and Biological Sciences, Abasyn University, Peshawar 25000, Pakistan.
3. Department of Natural Sciences, Lebanese American University, Beirut P.O. Box 36, Lebanon.
4. Gilbert and Rose-Marie Chagoury School of Medicine, Lebanese American University, Beirut P.O. Box 36, Lebanon.
5. Department of Chemistry, College of Arts and Sciences, IUBAT-International University of Business Agriculture and Technology, Dhaka 1230, Bangladesh.
6. Center for Global Health Research, Saveetha Institute of Medical and Technical Sciences in Saveetha Medical College and Hospital, Chennai, India.
7. Department of Theoretical and Applied Chemistry, South Ural State University, Lenin prospect 76, Chelyabinsk, 454080, Russian Federation.

***Corresponding author: heba.hashem@women.asu.edu.eg**

Contents:

Experimental; chemistry, DFT (Molecular Optimization and evaluation of FMOs, and Chemical descriptors), *In-vitro* cytotoxic assay (MTT), Molecular docking interaction, IR , ^{1}H-NMR, ^{13}C-NMR, and Mass spectra of the new compounds.

3. Experimental

3.1. Chemistry

All melting points were determined on a Gallenkamp apparatus and are uncorrected. The IR spectra were measured on a Pye-UnicamSP300 instrument in potassium bromide discs. The ^{1}H-NMR and ^{13}C-NMR spectra were recorded on Varian Mercury VX (400 MHz) spectrometer (with operating frequencies 500 MHz for 1H using TMS as an internal standard and 125 MHz for 13C). Chemical shifts (δ) are reported in parts per million (ppm) and coupling constants (J) are reported in Hertz (Hz). NMR spectra were referenced to the residual signals of DMSO-d6. Mass spectra were run on a MAT Finnigan SSQ 7000 spectrometer, using the electron impact technique (EI). Elemental analyses were carried out by the Micro analytical Center of Cairo University, Giza, Egypt. The progression of the reactions was monitored using TLC Merck Kieselgel 60 F254 aluminum packed plates.

3.2.Molecular Optimization and evaluation of FMOs, and Chemical descriptors

Structure optimization and DFT was permed by material studio version 8.0 with the DMol3 and hybrid functional B3LYP . The following equation was used to calculate the chemical reactivity descriptor including chemical energy gap (E_{gap}), chemical potentiality (μ), hardness (η), softness (S), and electronegativity (χ).

$E_{gap} = E_{HOMO} – E_{LUMO}$…………………… (1)

$I = – E_{HOMO}$ ……………………………… (2)

$A = – E_{LUMO}$ …………………………... (3)

$\mu = – (1+A)/2$ …………………………… (4)

$\eta = (1–A)/2$……………………………… (5)

$S = 1/\eta$ …………………………………… (6)

$\chi = (1+A)/2$ ……………………………… (7)

$\omega = \mu^2/2\eta$ ………………………………… (8)

3.3.In Vitro MTT cytotoxicity assay

To assess the half maximal cytotoxic concentration (CC50), stock solutions of the test compounds were prepared in 10 % DMSO in ddH2O and diluted further to the working solutions with DMEM. The cytotoxic activity of the extracts was tested in VERO-E6 cells by using the 3-(4, 5-dimethylthiazol -2-yl)-2, 5-diphenyltetrazolium bromide (MTT) method with minor modifications. Briefly, the cells were seeded in 96 well-plates (100 µl/well at a density of 3×105 cells/ml) and incubated for 24 h at 37 °C in 5%CO2. After 24 h, cells were treated with various concentrations of the tested compounds in triplicates. 24 h later, the supernatant was discarded, and cell monolayers were washed with sterile 1x phosphate buffer saline (PBS) 3 times and MTT solution (20 µl of 5 mg/ml stock solution) was add to each well and incubated at 37 °C for 4 h followed by medium aspiration. In each well, the formed formazan crystals were dissolved with 200 µl of acidified isopropanol (0.04 M HCl in absolute isopropanol = 0.073 ml HCL in 50 ml isopropanol). Absorbance of formazan solutions was measured at λ max 540 nm with 620 nm as a reference wavelength using a multi-well plate reader. The percentage of cytotoxicity compared to the untreated cells was determined with the following equation.

The plot of % cytotoxicity versus sample concentration was used to calculate the concentration which exhibited 50% cytotoxicity (CC50).

% cytotoxicity = ((absorbance of cells without treatment-absorbance of cells with treatment) / (absorbance of cells without treatment) X 100)

3.4.Inhibitory concentration 50 (IC_{50}) determination

In 96-well tissue culture plates, 2.4×10^4 Vero-E6 cells were distributed in each well and incubated overnight at a humidified 37°C incubator under 5%CO2 condition. The cell monolayers were then washed once with 1x PBS and subjected to virus adsorption (hCoV-19/Egypt/NRC-03/2020 (Accession Number on GSAID: EPI_ISL_430820)) for 1 h at room temperature (RT). The cell monolayers were further overlaid with 100µl of DMEM containing varying concentrations of the test compounds. Following incubation at 37°C in 5% CO2 incubator for 72 h, the cells were fixed with 100 µl of 4% paraformaldehyde for 20 min and stained with 0.1% crystal violet in distilled water for 15 min at RT. The crystal violet dye was then dissolved using 100 µl absolute methanol per well and the optical density of the color is measured at 570 nm using Anthos Zenyth 200rt plate reader (Anthos Labtec Instruments, Heerhugowaard, Netherlands). The IC_{50} of the compound is that required to reduce the virus-induced cytopathic effect (CPE) by 50%, relative to the virus control.

Molecular docking

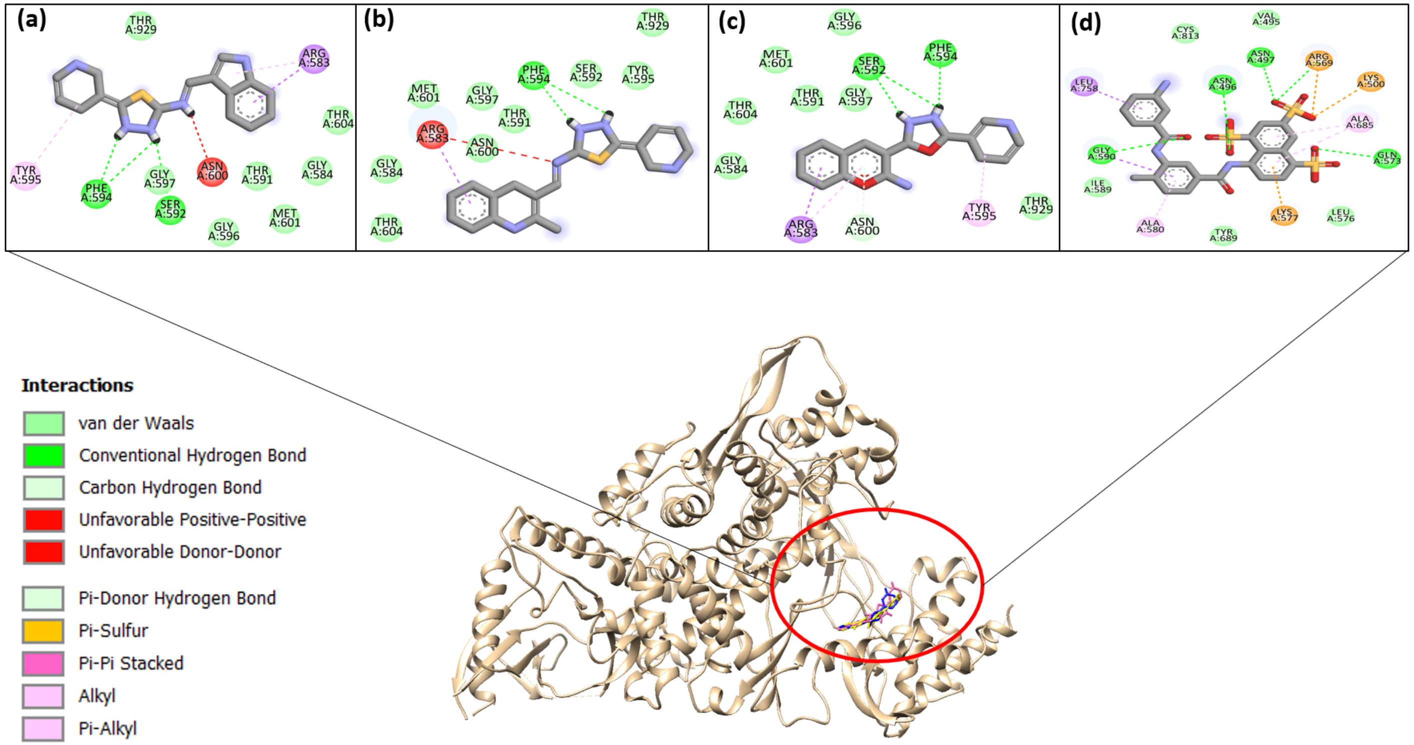

S-Fig.1. Compounds docked conformation with SARS-CoV-2 RdRP enzyme. The enzyme is shown tan cartoon, while compounds are in different colors stick. The **11**, **12** and **5** is in yellow, pink and blue stick, respectively. The interactions of **11** (a), **12** (b), **5** (c) and **control** (d) are also given.

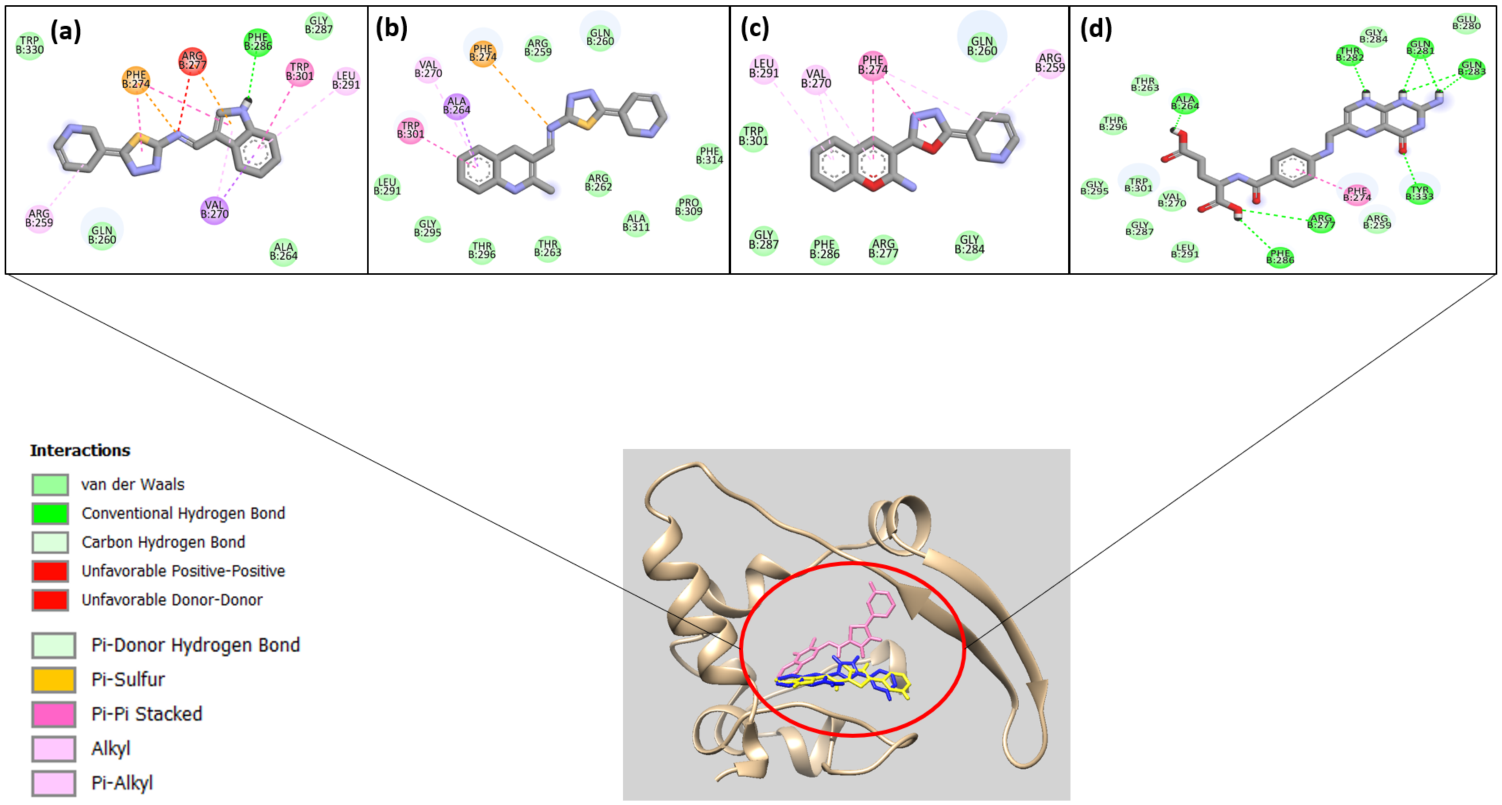

S-Fig.2. Compounds docked conformation with SARS-CoV-2 Nucleocapsid protein. The enzyme is shown tan cartoon, while compounds are in different colors stick. The **11**, **12** and **5** is in yellow, pink and blue stick, respectively. The interactions of **11** (a), **12** (b), **5** (c) and **control** (d) are also given.

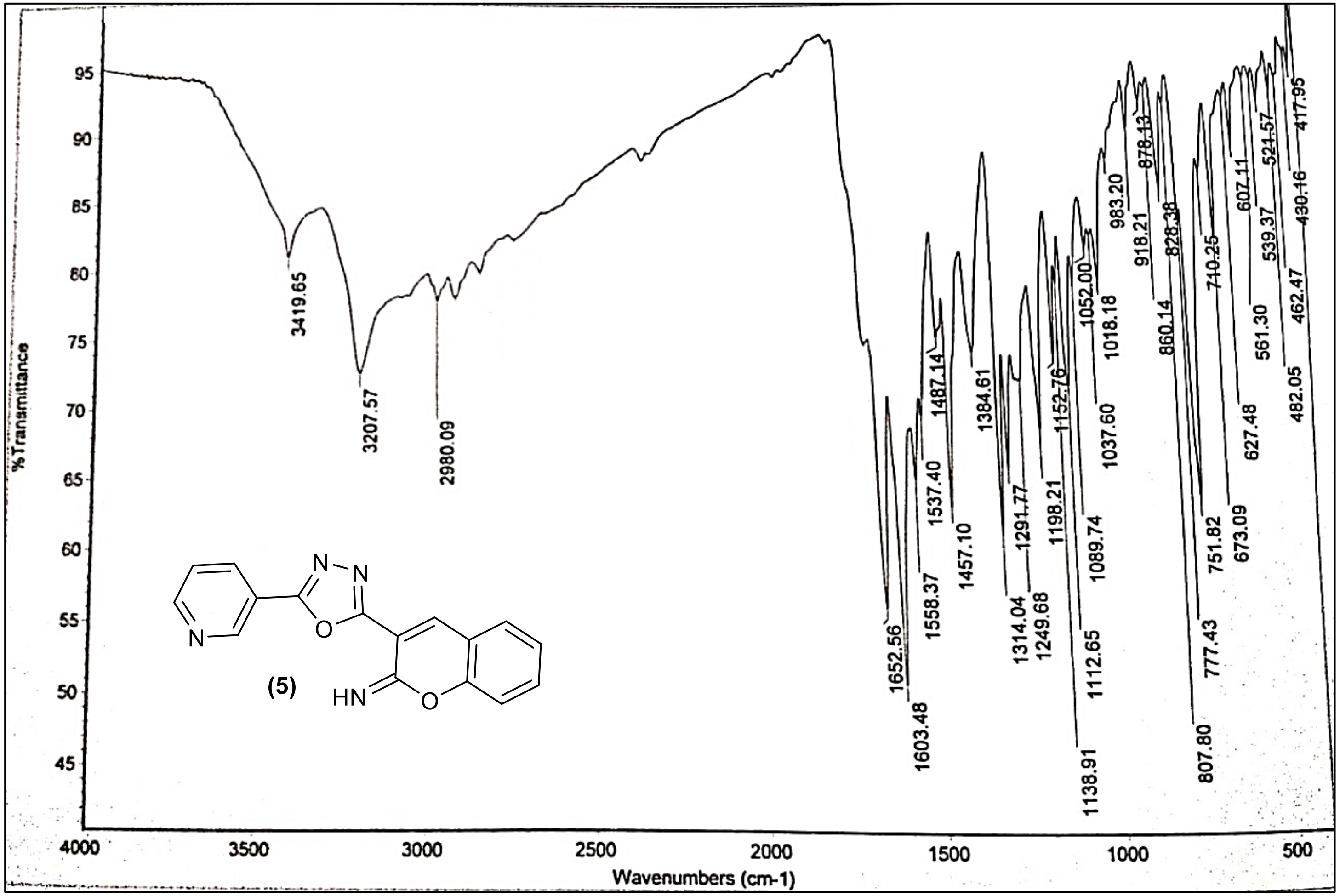

IR spectrum of *3-(5-(pyridin-3-yl)-1,3,4-oxadiazol-2-yl)-2H-chromen-2-imine* ***(5)***

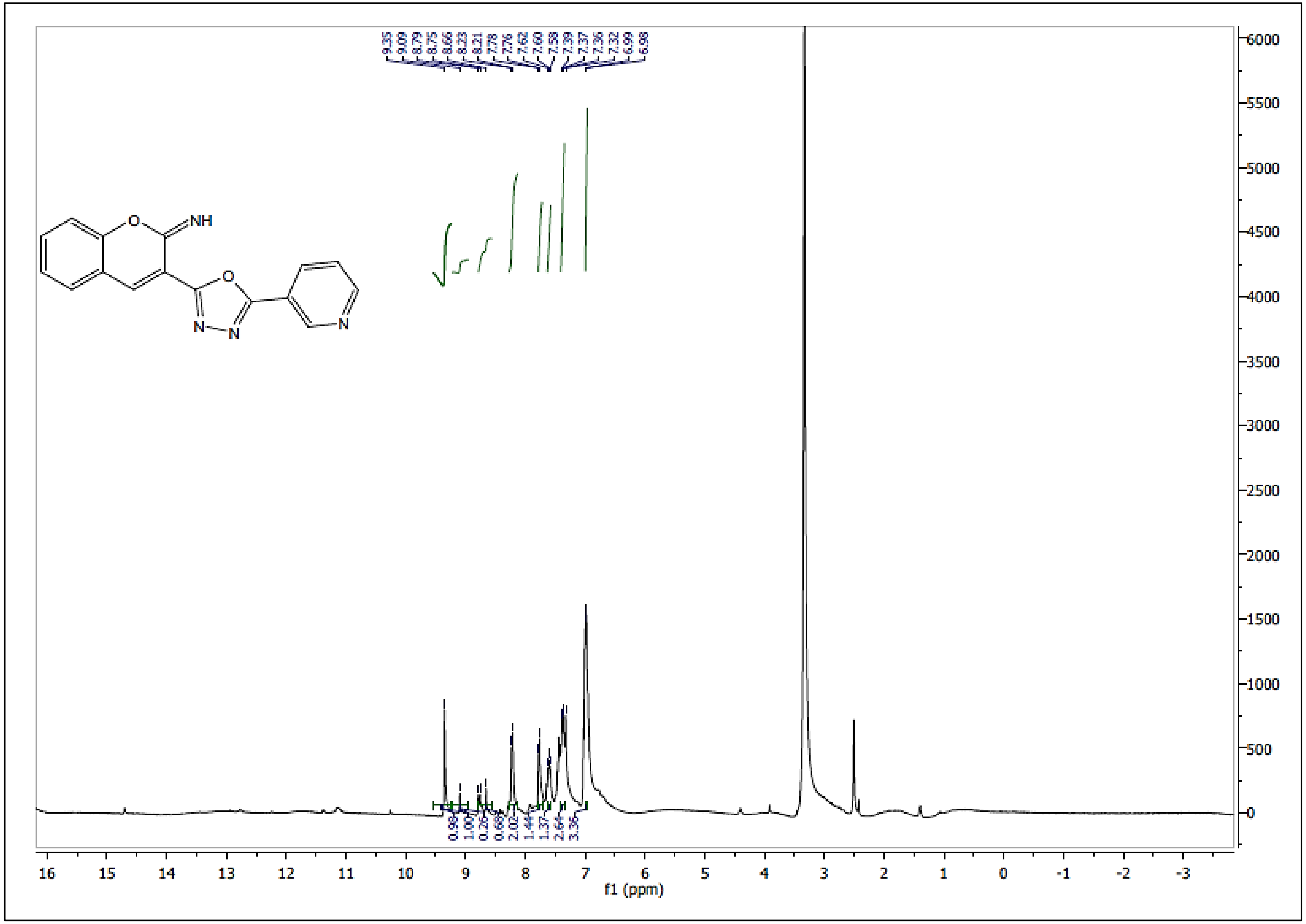

^{1}HNMR spectrum of *3-(5-(pyridin-3-yl)-1,3,4-oxadiazol-2-yl)-2H-chromen-2-imine* ***(5)***

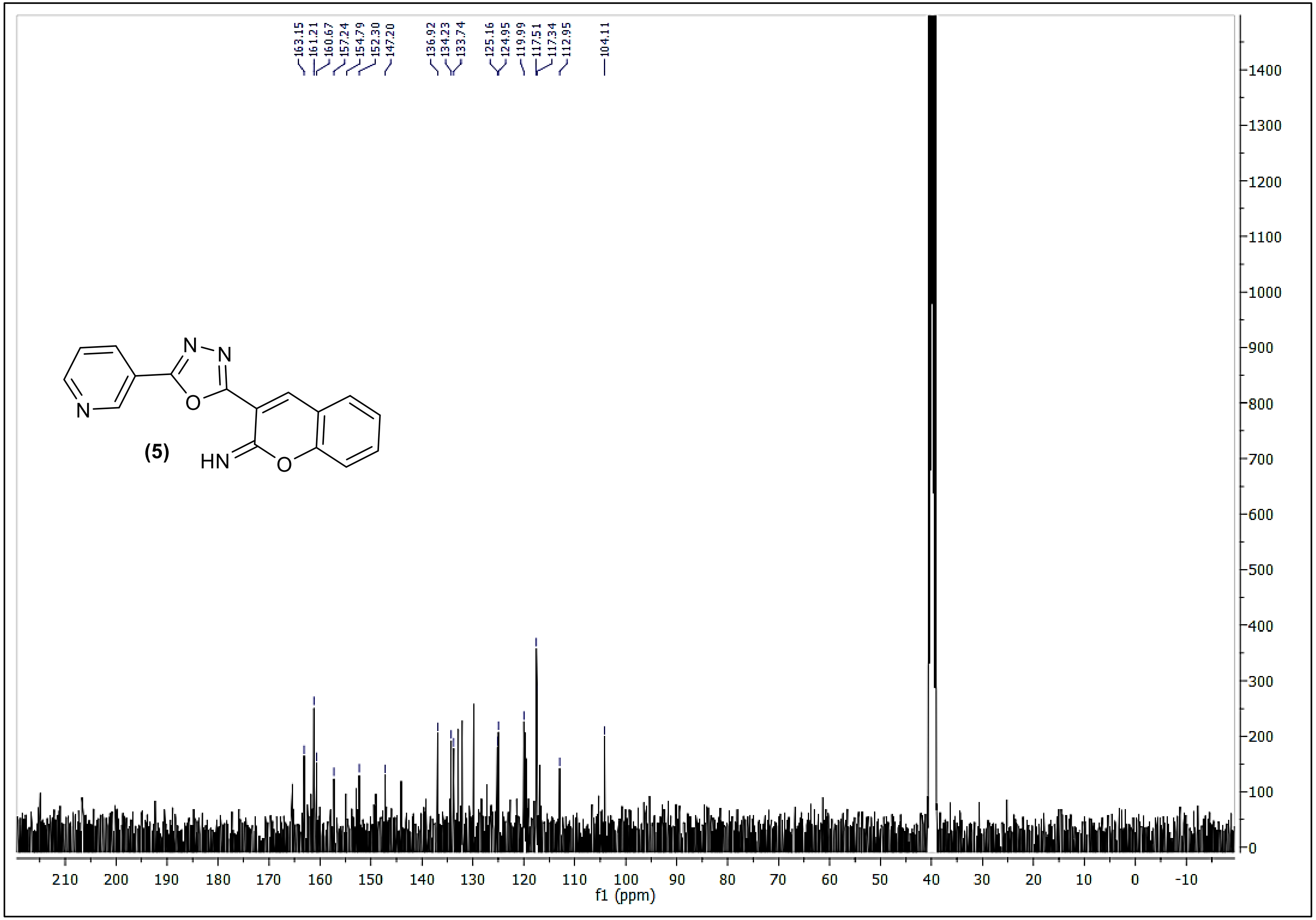

^{13}CNMR spectrum of *3-(5-(pyridin-3-yl)-1,3,4-oxadiazol-2-yl)-2H-chromen-2-imine* ***(5)***

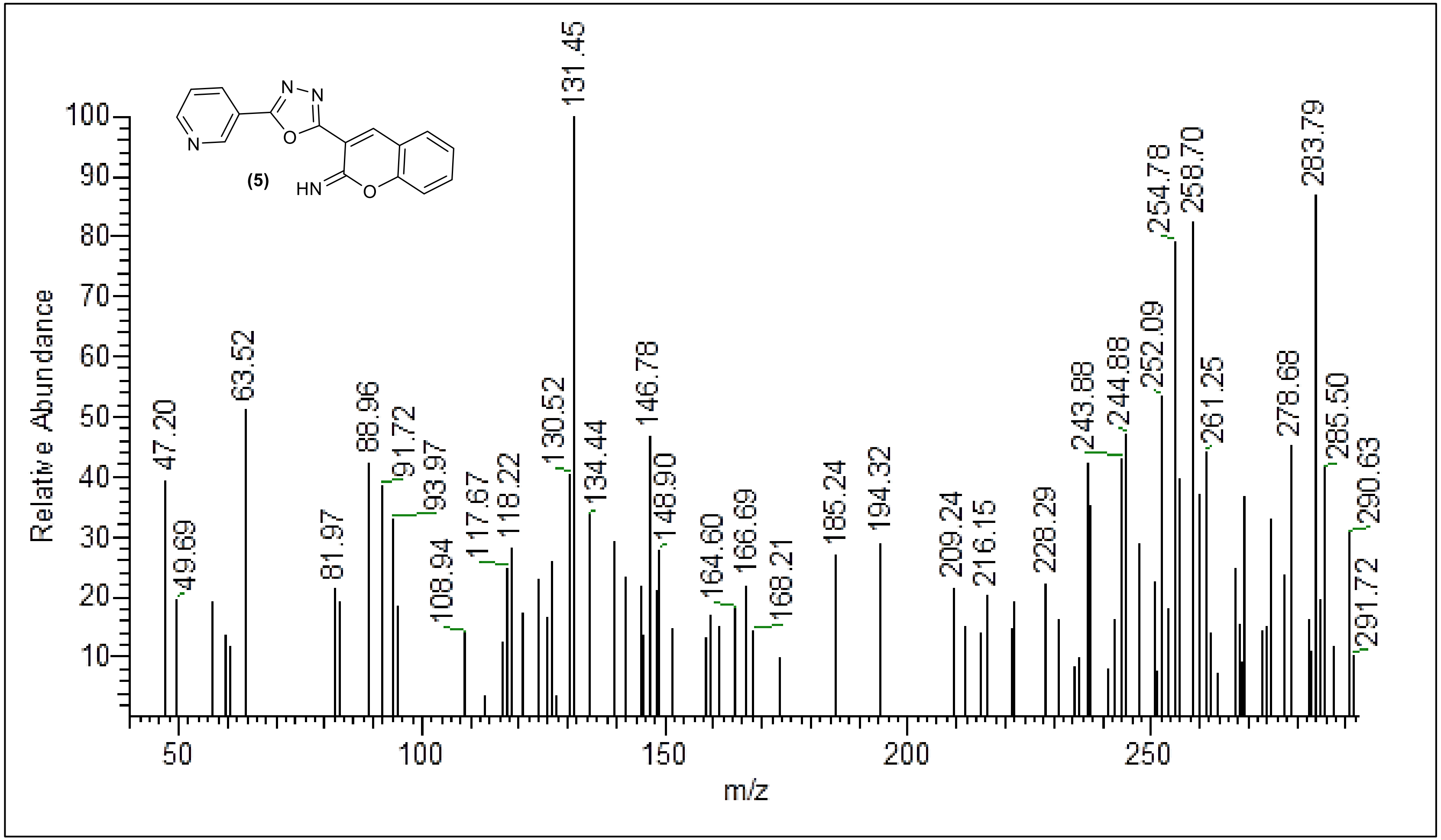

Mass spectrum of *3-(5-(pyridin-3-yl)-1,3,4-oxadiazol-2-yl)-2H-chromen-2-imine* ***(5)***

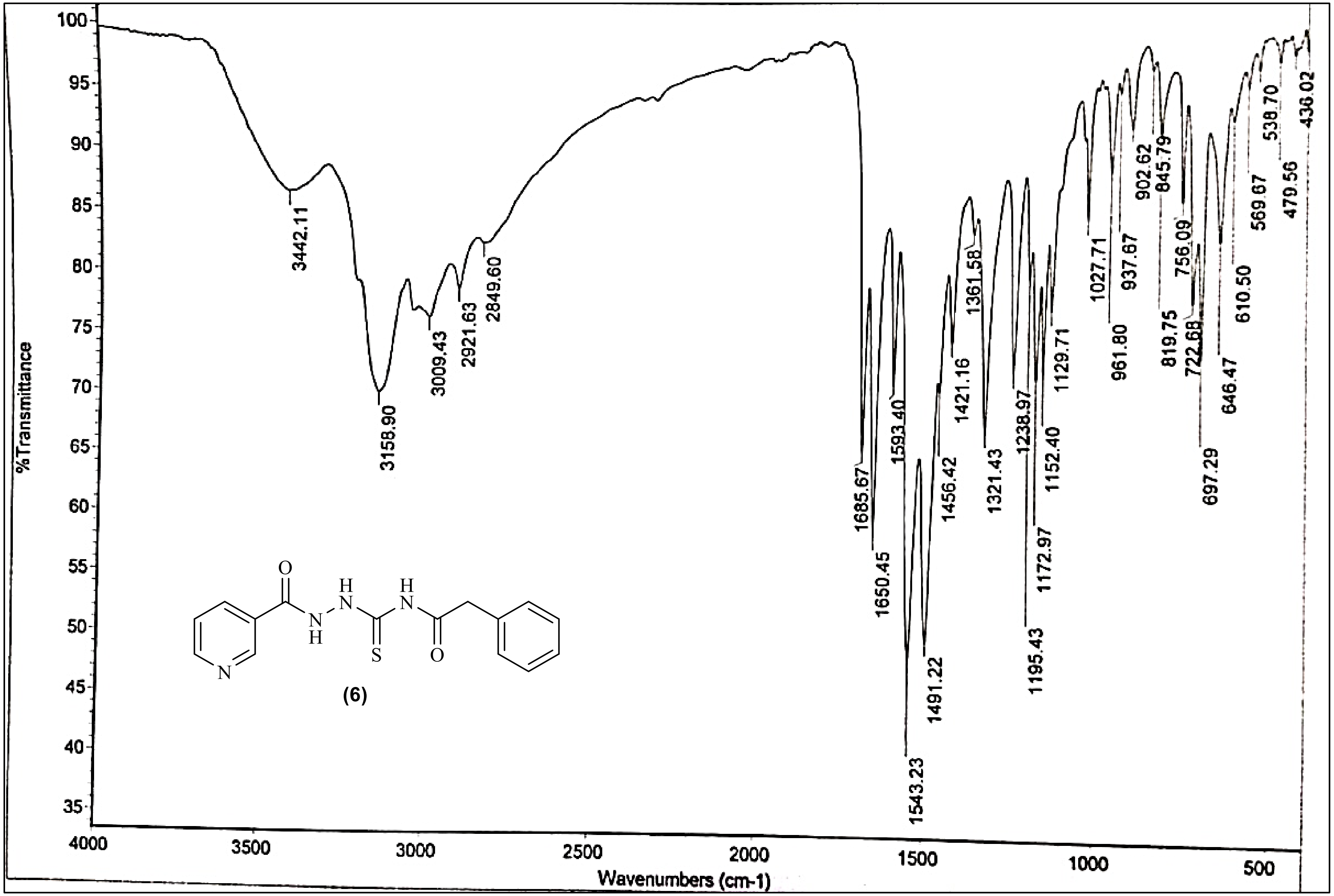

IR spectrum of *N-(2-nicotinoylhydrazine-1-carbonothioyl)-2-phenylacetamide* **(6)**

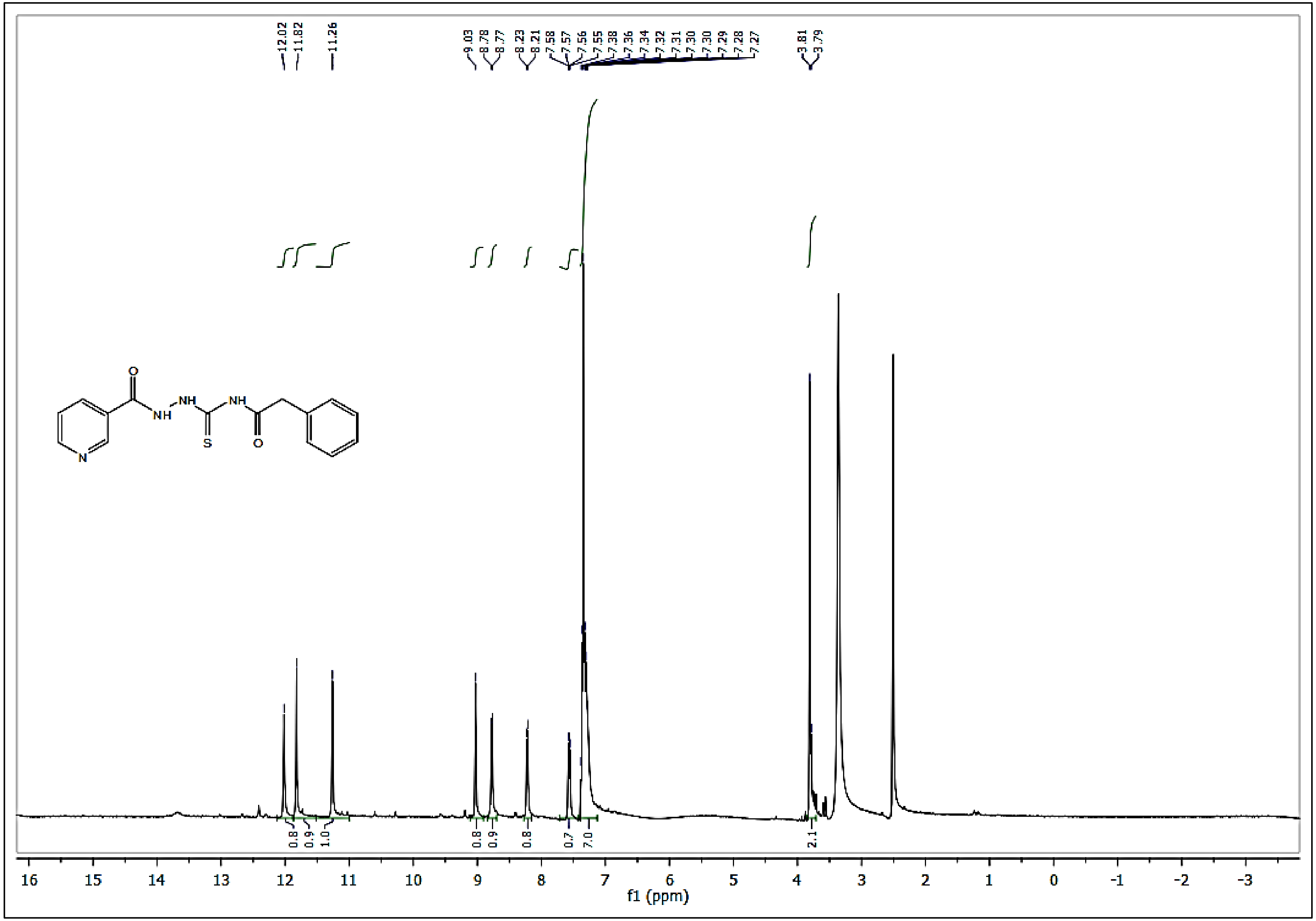

^{1}HNMR spectrum of *N-(2-nicotinoylhydrazine-1-carbonothioyl)-2-phenylacetamide* **(6)**

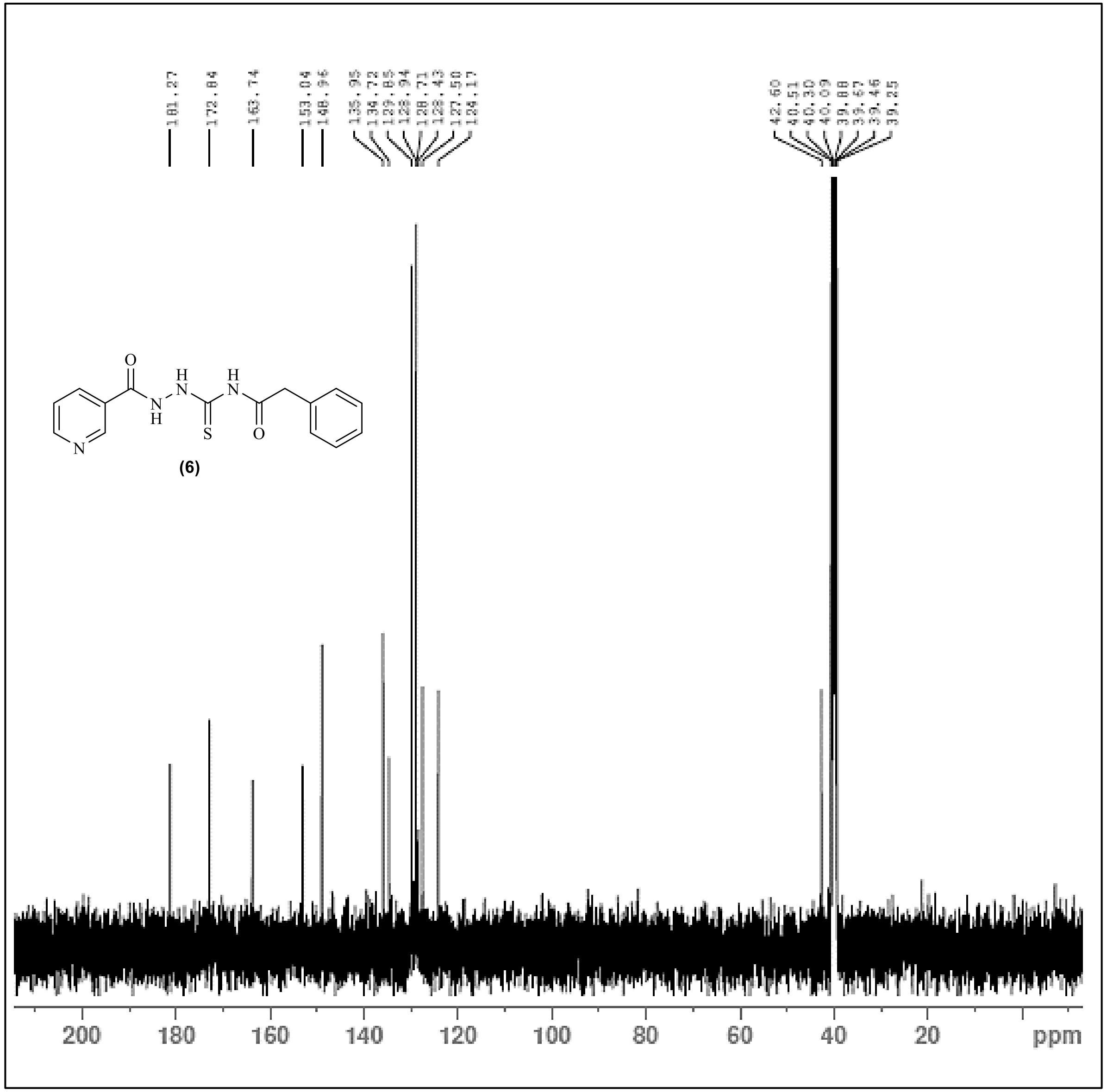

^{13}CNMR spectrum of *N-(2-nicotinoylhydrazine-1-carbonothioyl)-2-phenylacetamide* **(6)**

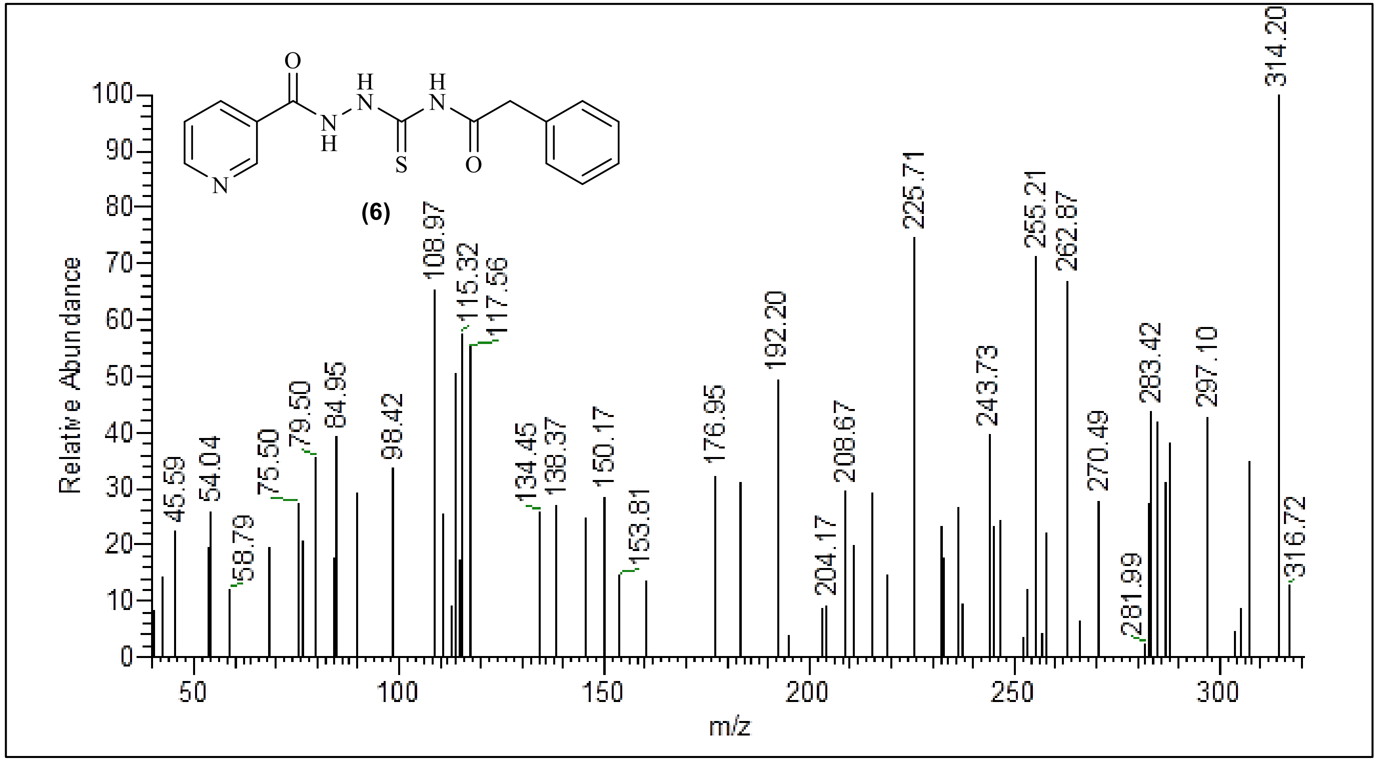

Mass spectrum of *N-(2-nicotinoylhydrazine-1-carbonothioyl)-2-phenylacetamide* **(6)**

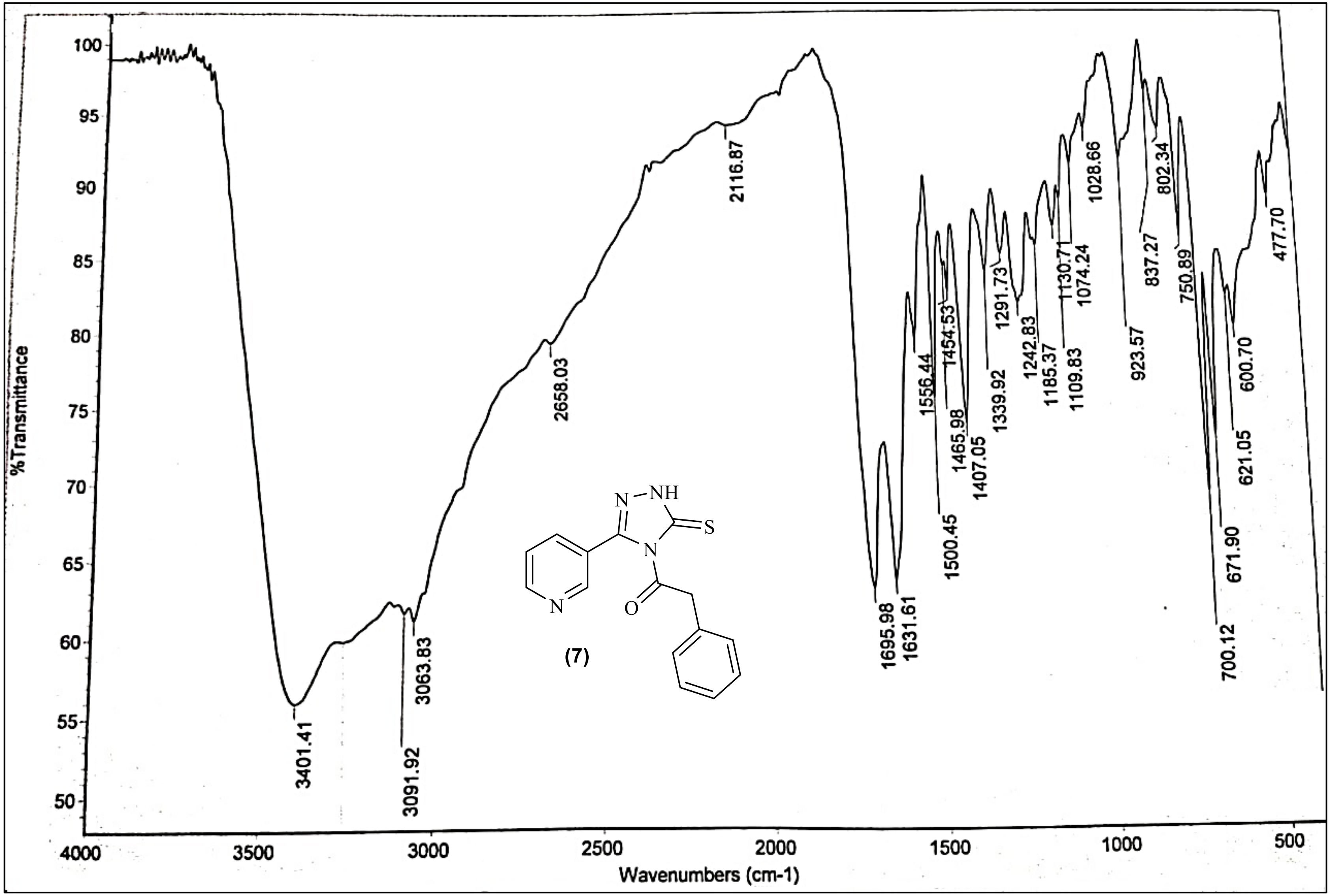

IR spectrum of *2-phenyl-1-(3-(pyridin-3-yl)-5-thioxo-1,5-dihydro-4H-1,2,4-triazol-4-yl)ethan-1-one* ***(7):***

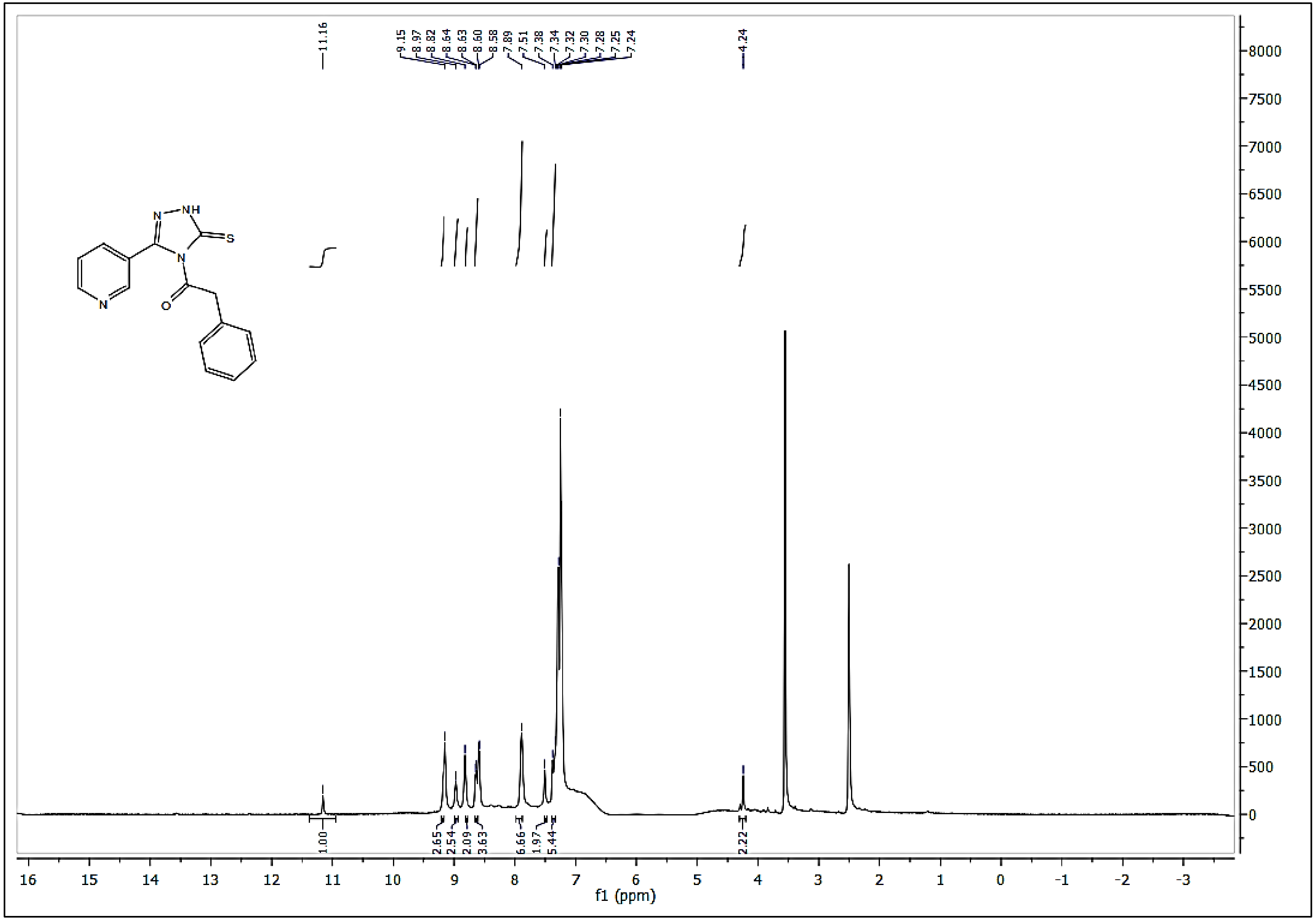

^{1}HNMR spectrum of *2-phenyl-1-(3-(pyridin-3-yl)-5-thioxo-1,5-dihydro-4H-1,2,4-triazol-4-yl)ethan-1-one* ***(7):***

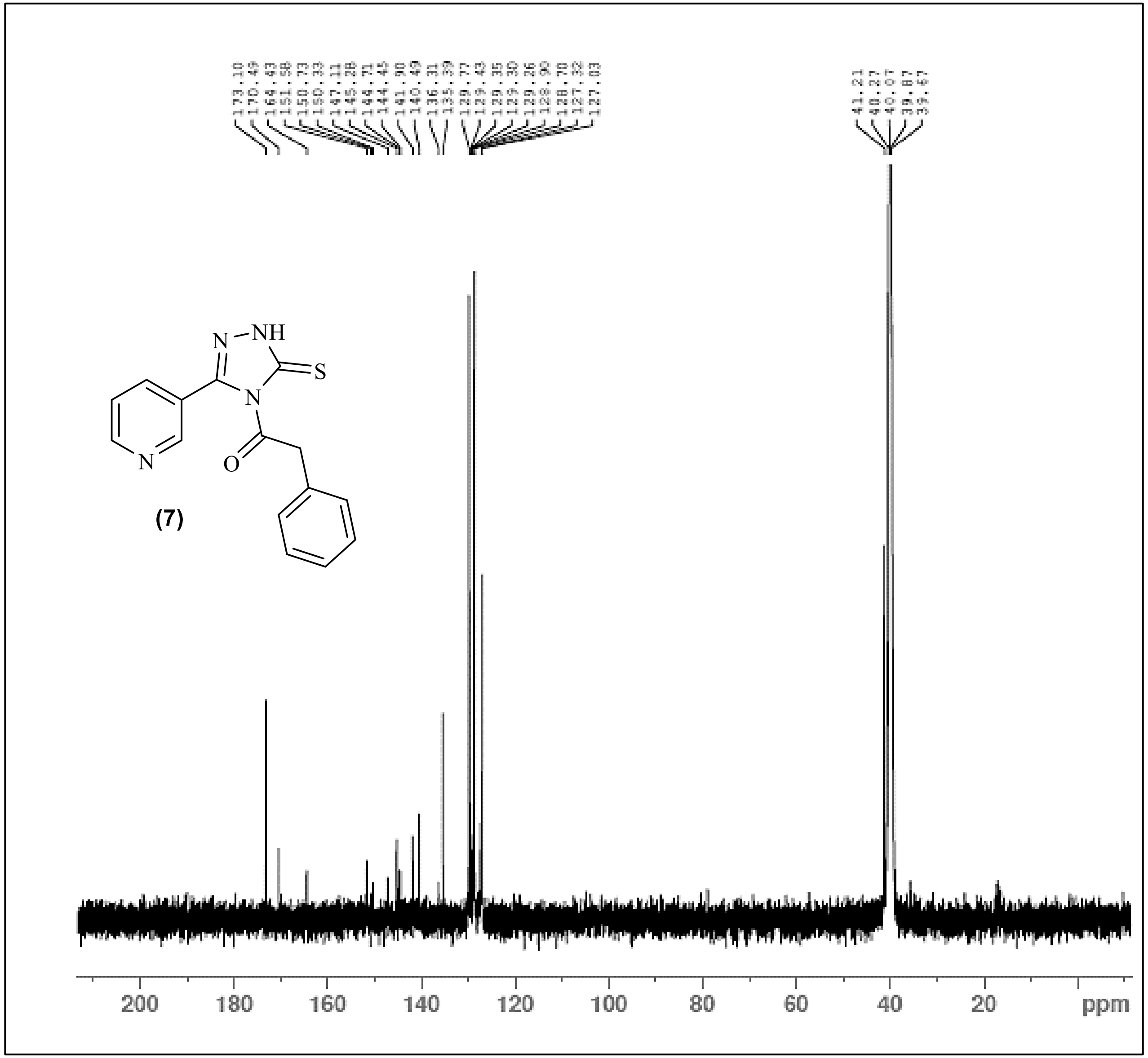

^{13}CNMR spectrum of *2-phenyl-1-(3-(pyridin-3-yl)-5-thioxo-1,5-dihydro-4H-1,2,4-triazol-4-yl)ethan-1-one* ***(7):***

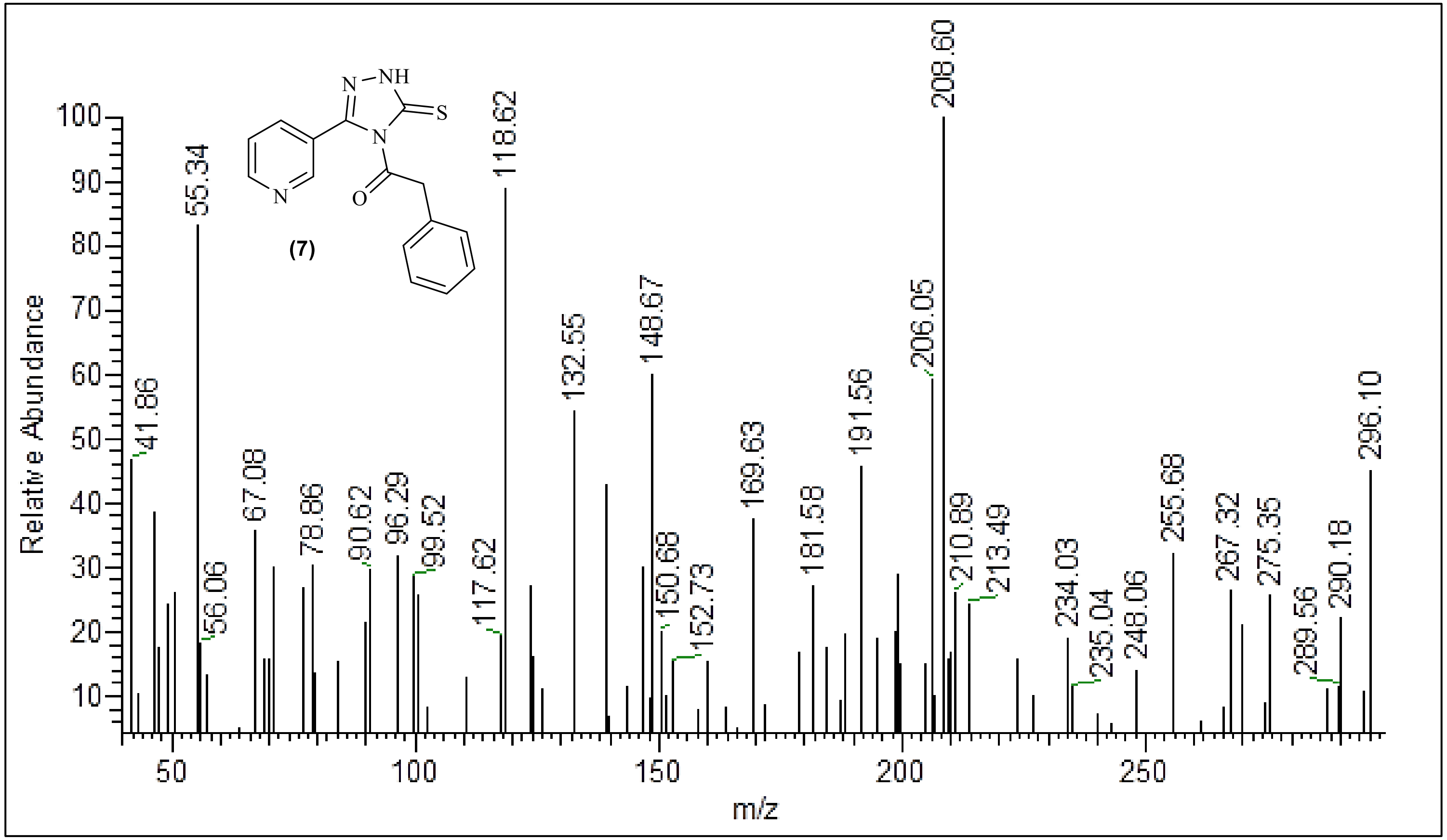

Mass spectrum of *2-phenyl-1-(3-(pyridin-3-yl)-5-thioxo-1,5-dihydro-4H-1,2,4-triazol-4-yl)ethan-1-one* ***(7):***

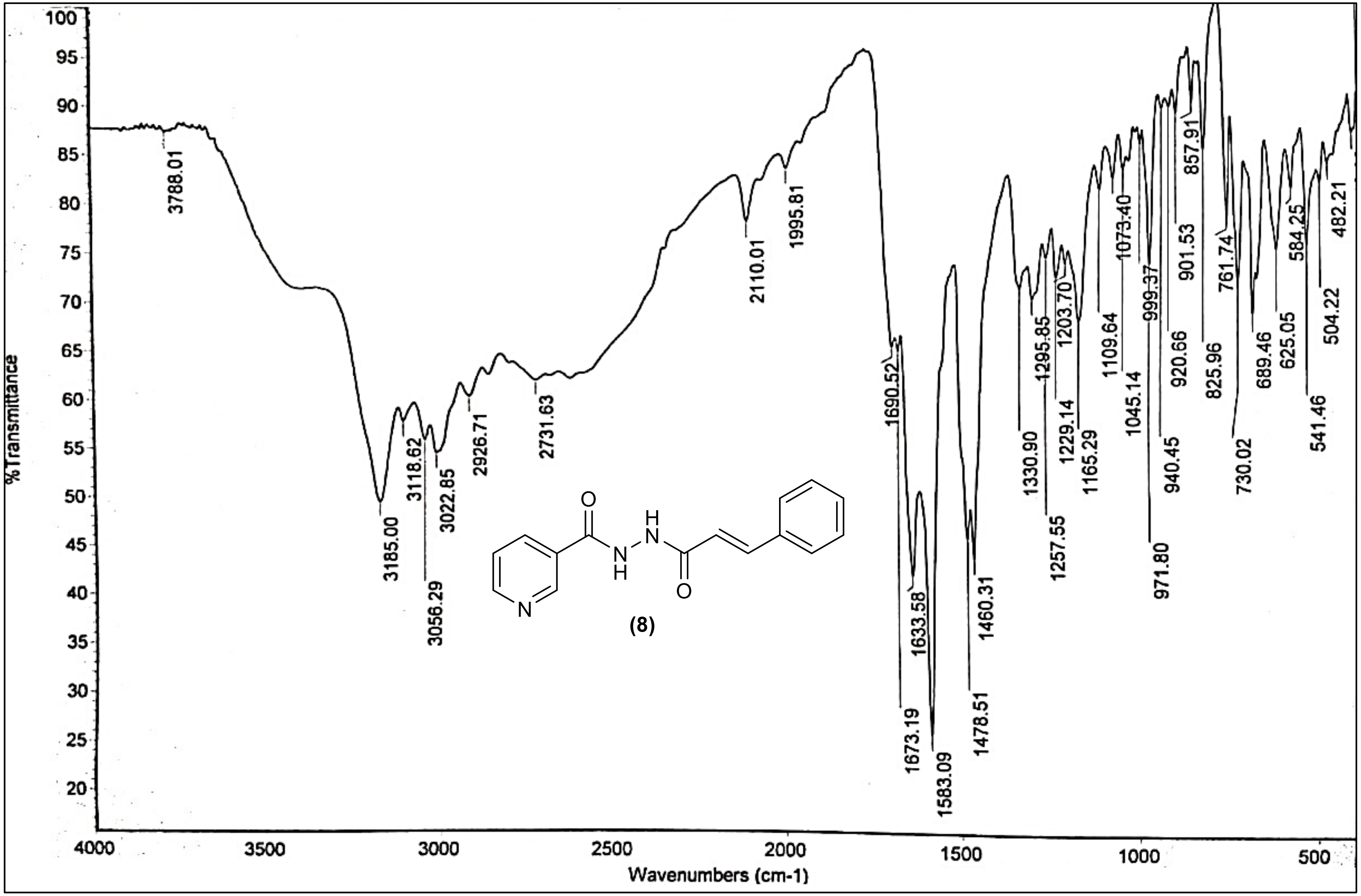

IR spectrum of *N'-cinnamoylnicotinohydrazide* ***8***

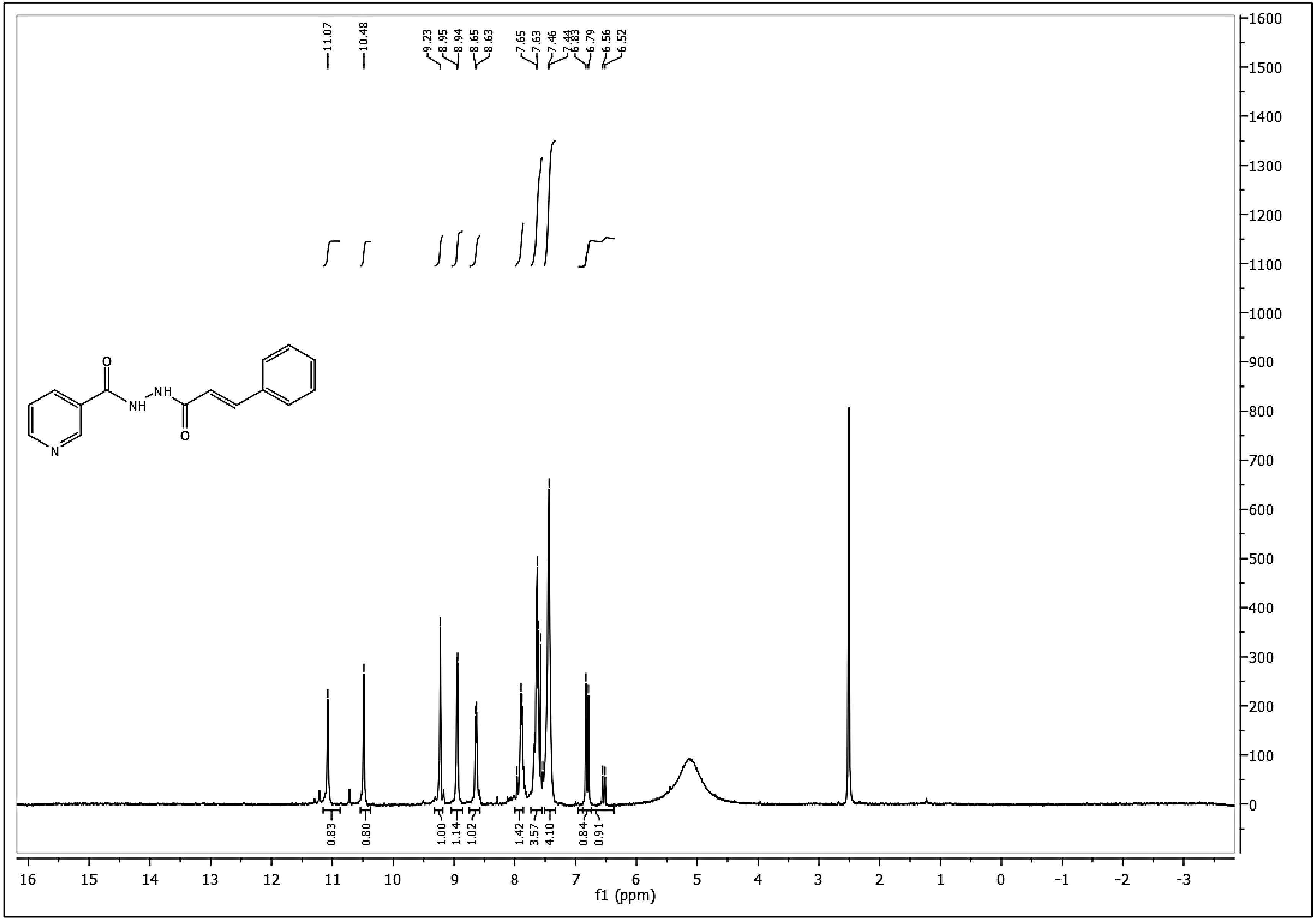

^{1}HNMR spectrum of *N'-cinnamoylnicotinohydrazide* ***8***

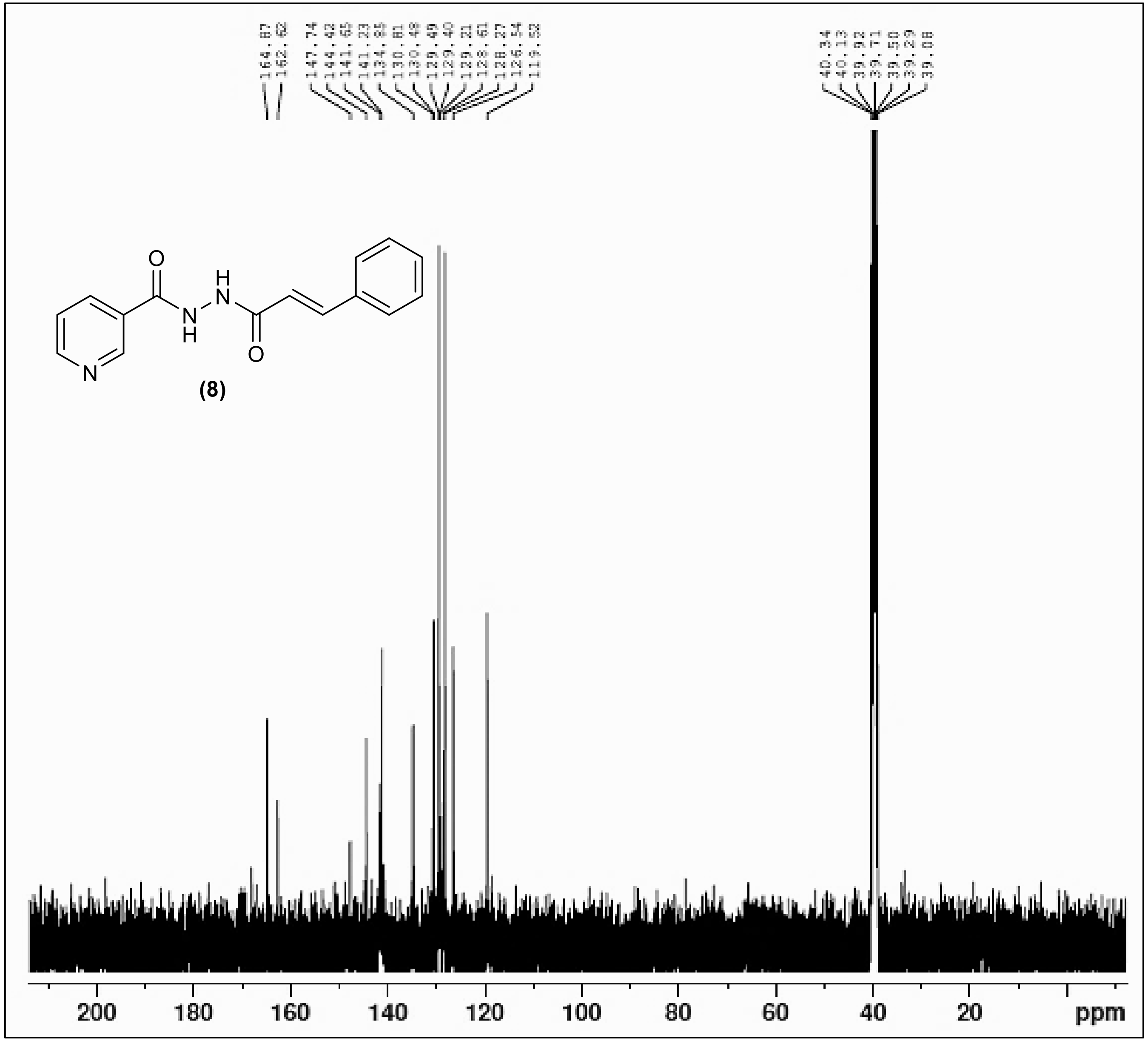

^{13}CNMR spectrum of *N'-cinnamoylnicotinohydrazide **8***

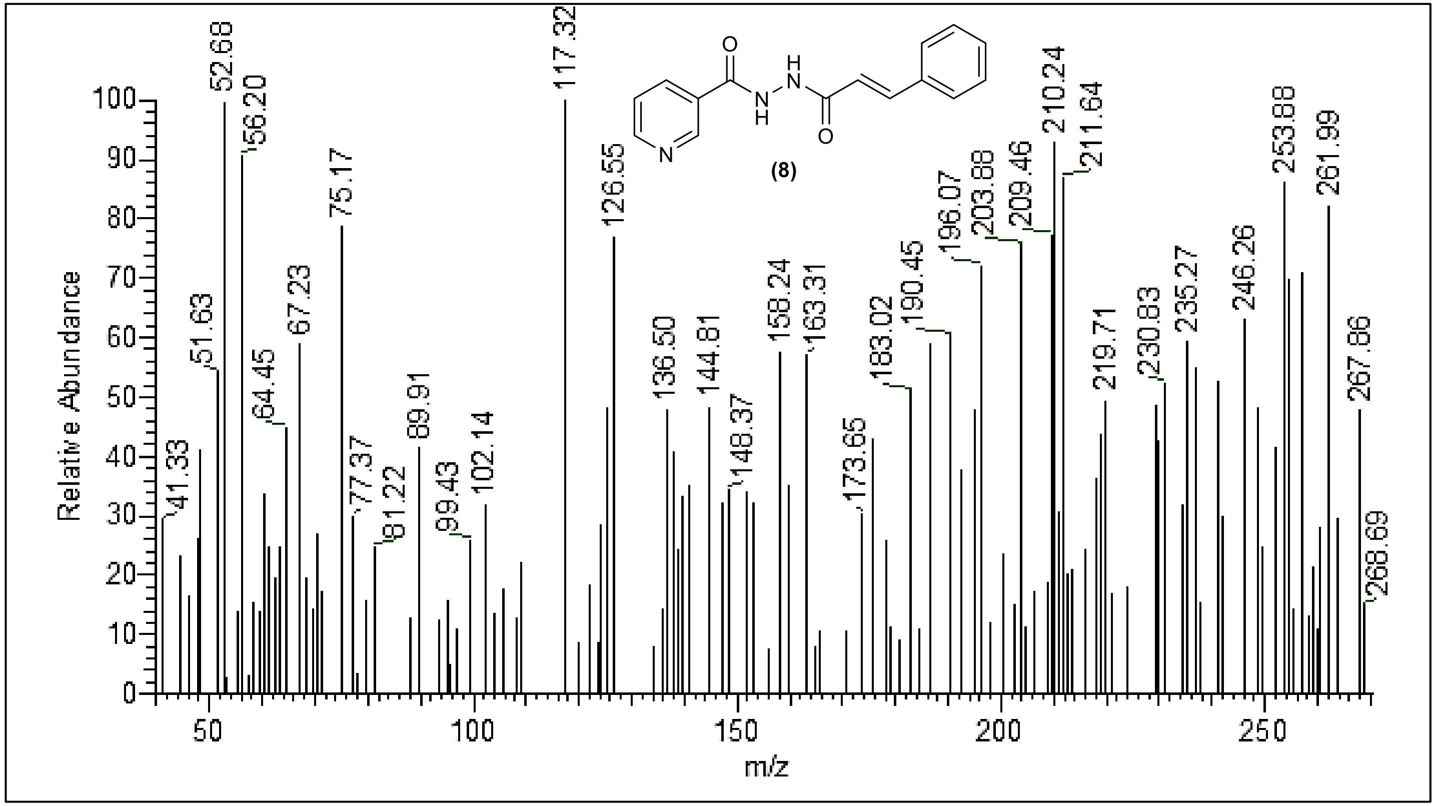

Mass spectrum of *N'-cinnamoylnicotinohydrazide **8***

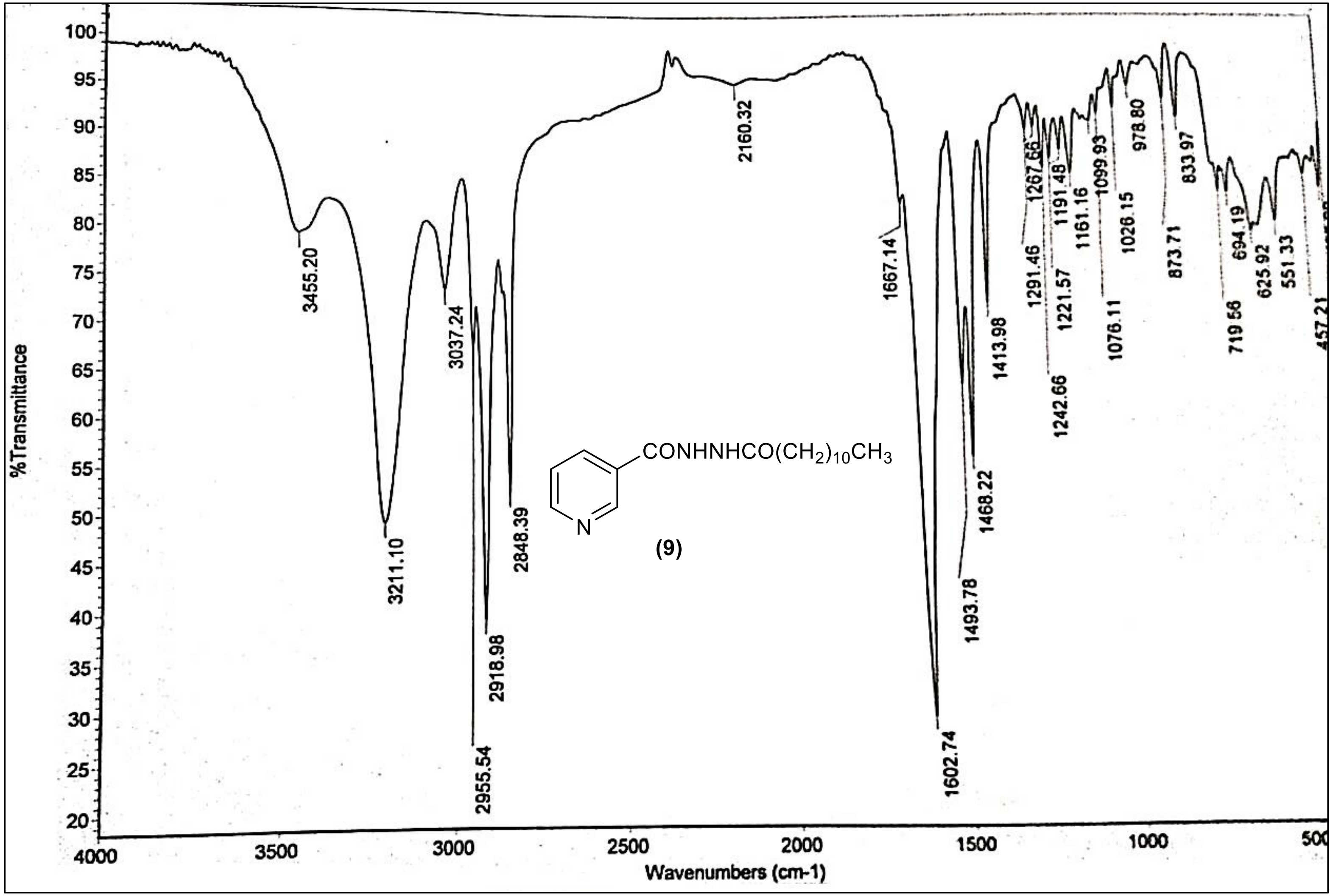

IR spectrum *of N'-dodecanoylnicotinohydrazide* **9**

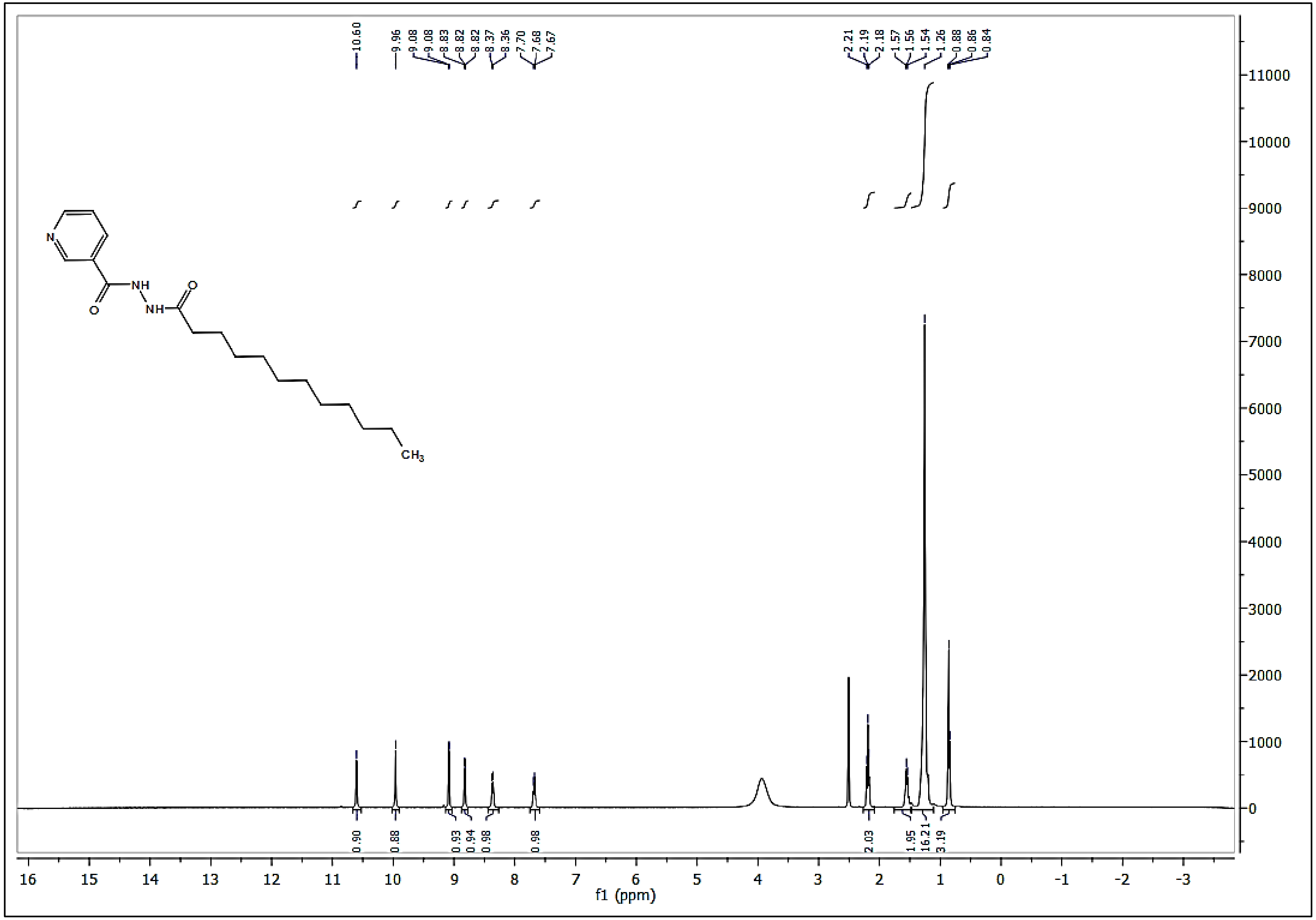

^{1}HNMR spectrum *of N'-dodecanoylnicotinohydrazide* **9**

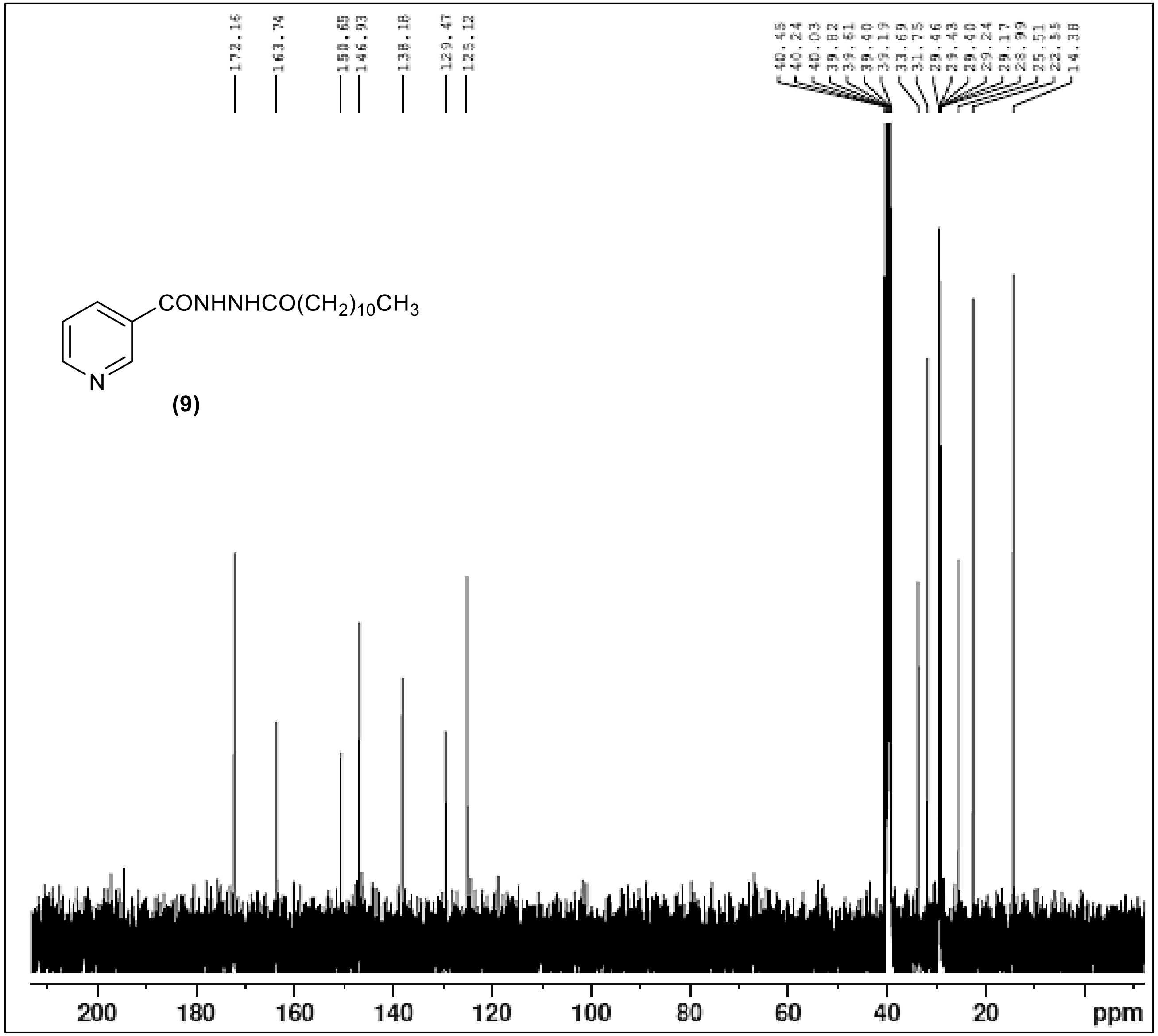

^{13}CNMR spectrum *of N'-dodecanoylnicotinohydrazide* **9**

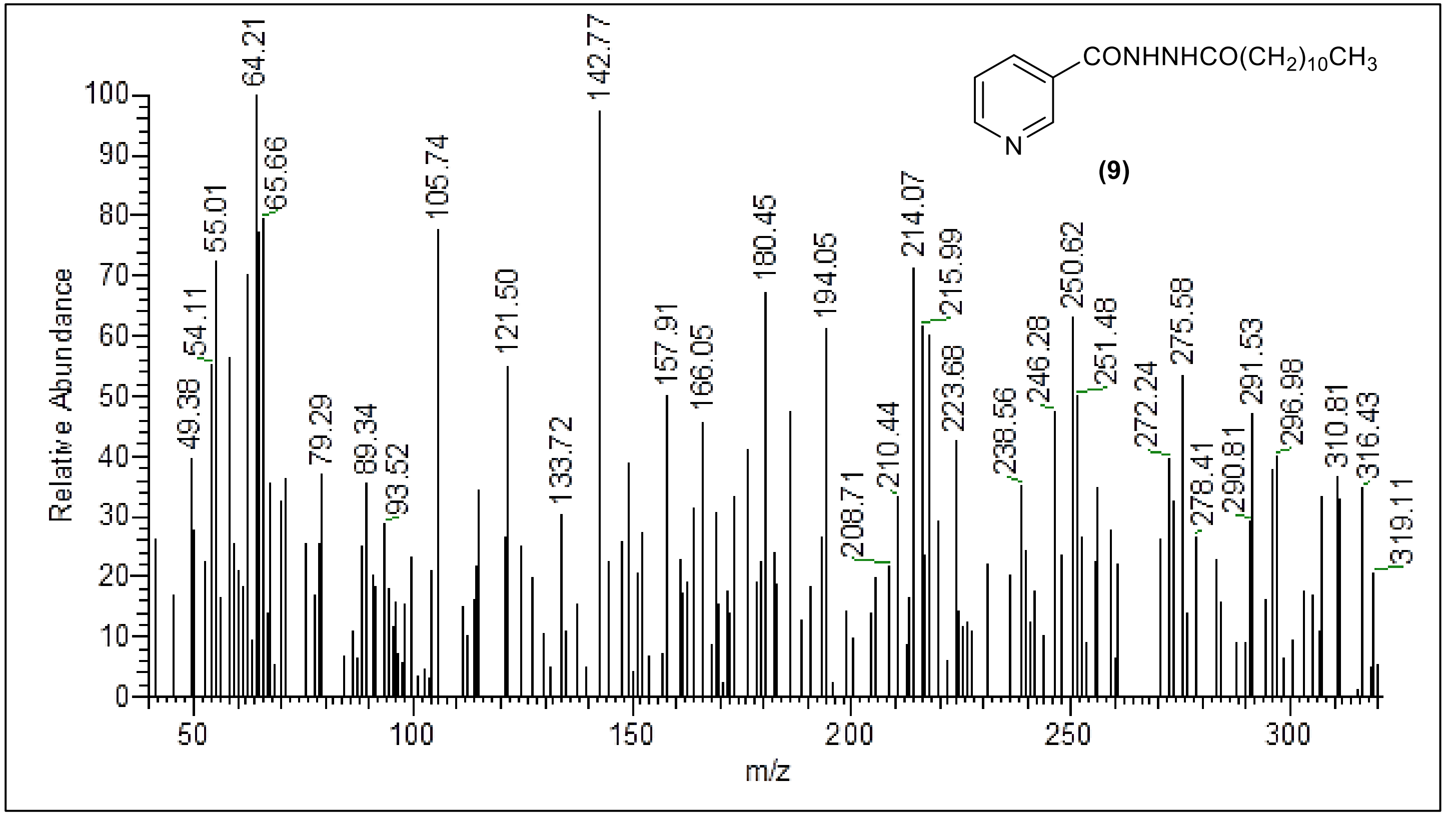

Mass spectrum *of N'-dodecanoylnicotinohydrazide* **9**

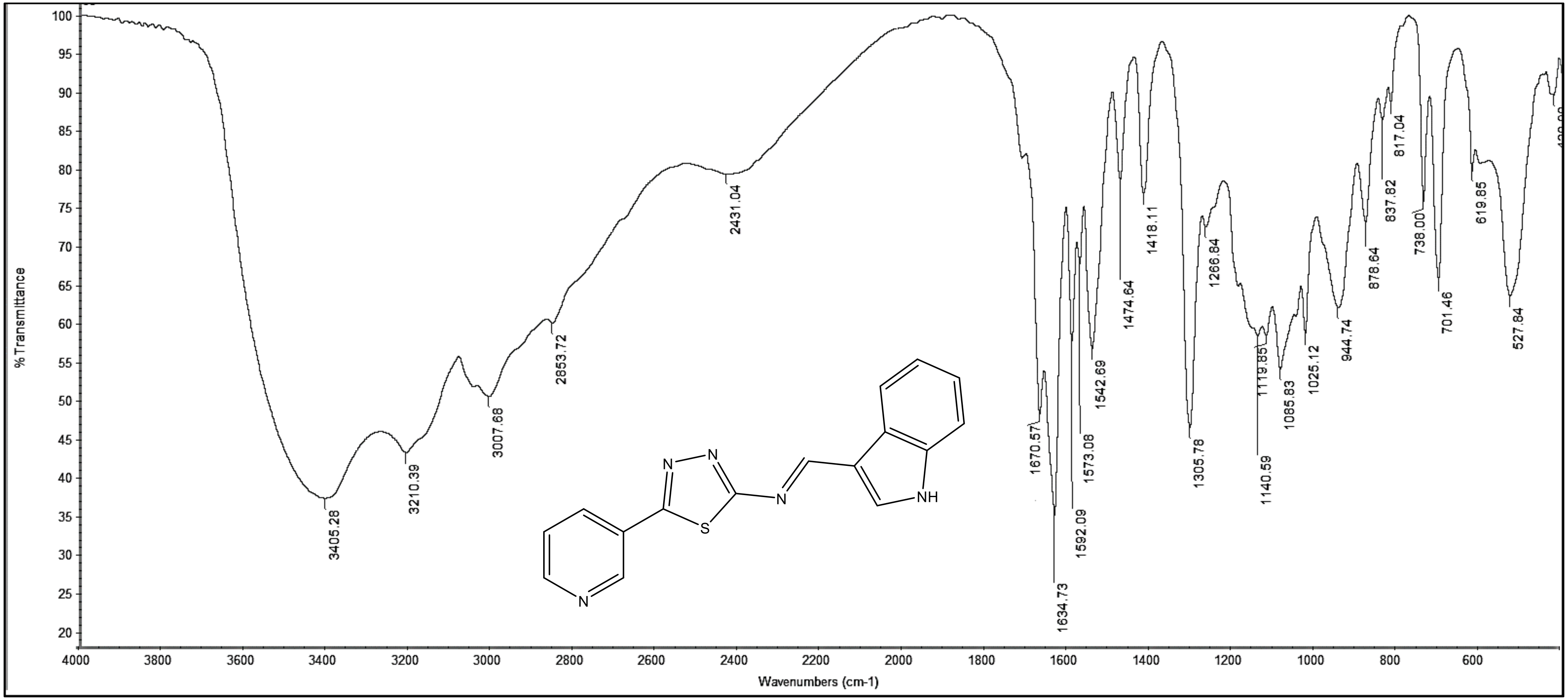

IR spectrum *of 1-(1H-indol-3-yl)-N-(5-(pyridin-3-yl)-1,3,4-thiadiazol-2-yl)methanimine* **11**

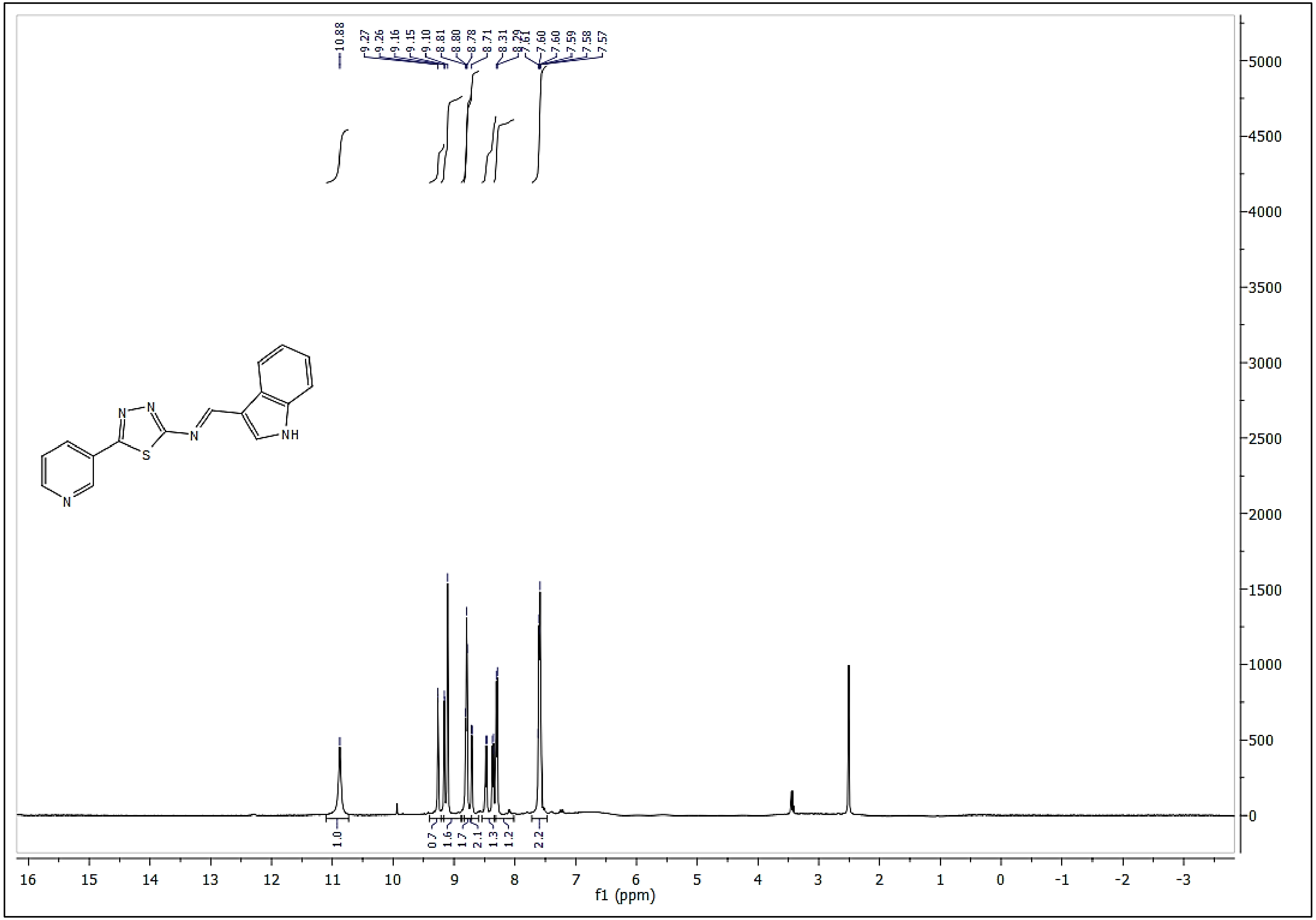

^{1}HNMR spectrum *of 1-(1H-indol-3-yl)-N-(5-(pyridin-3-yl)-1,3,4-thiadiazol-2-yl)methanimine* **11**

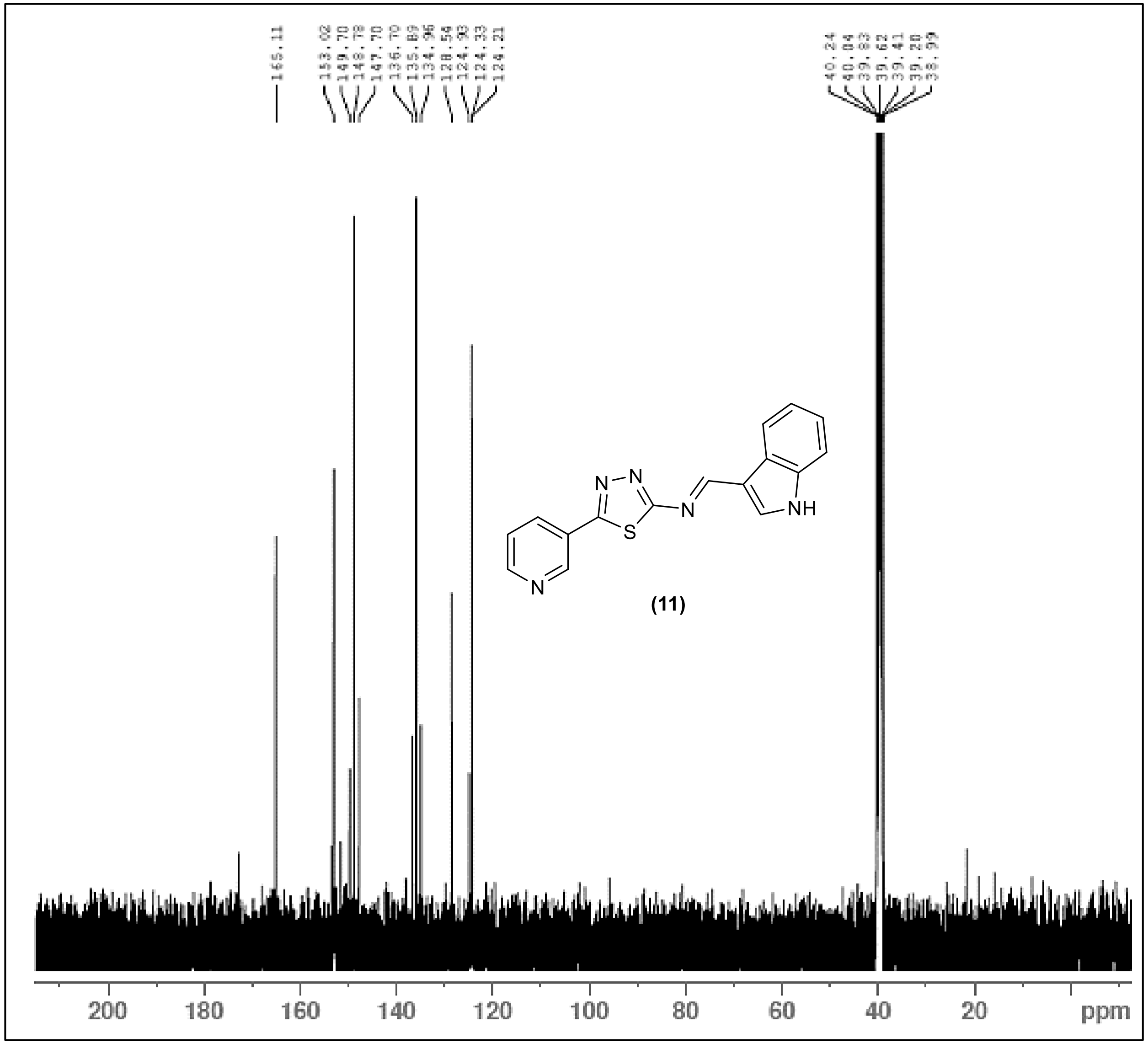

^{13}CNMR spectrum *of 1-(1H-indol-3-yl)-N-(5-(pyridin-3-yl)-1,3,4-thiadiazol-2-yl)methanimine* **11**

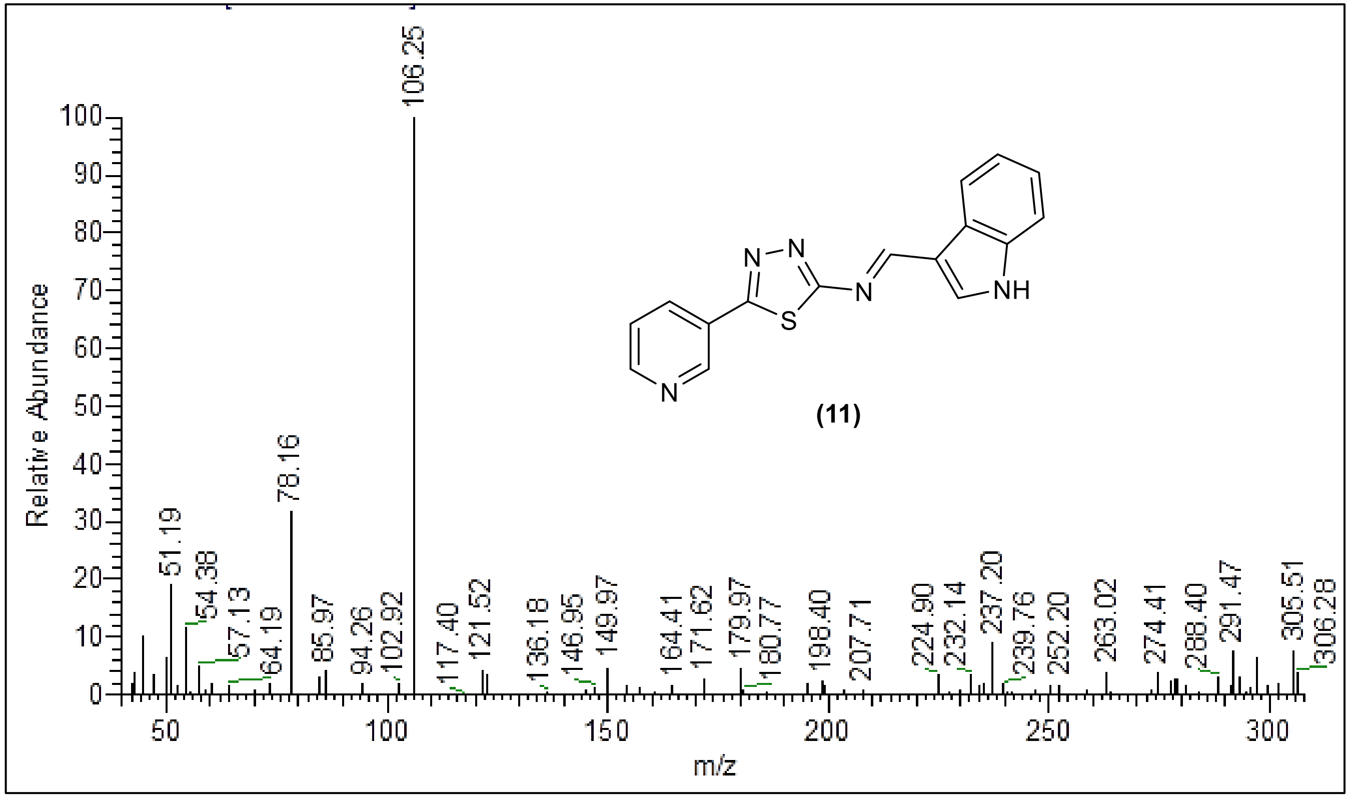

Mass spectrum *of 1-(1H-indol-3-yl)-N-(5-(pyridin-3-yl)-1,3,4-thiadiazol-2-yl)methanimine* **11**

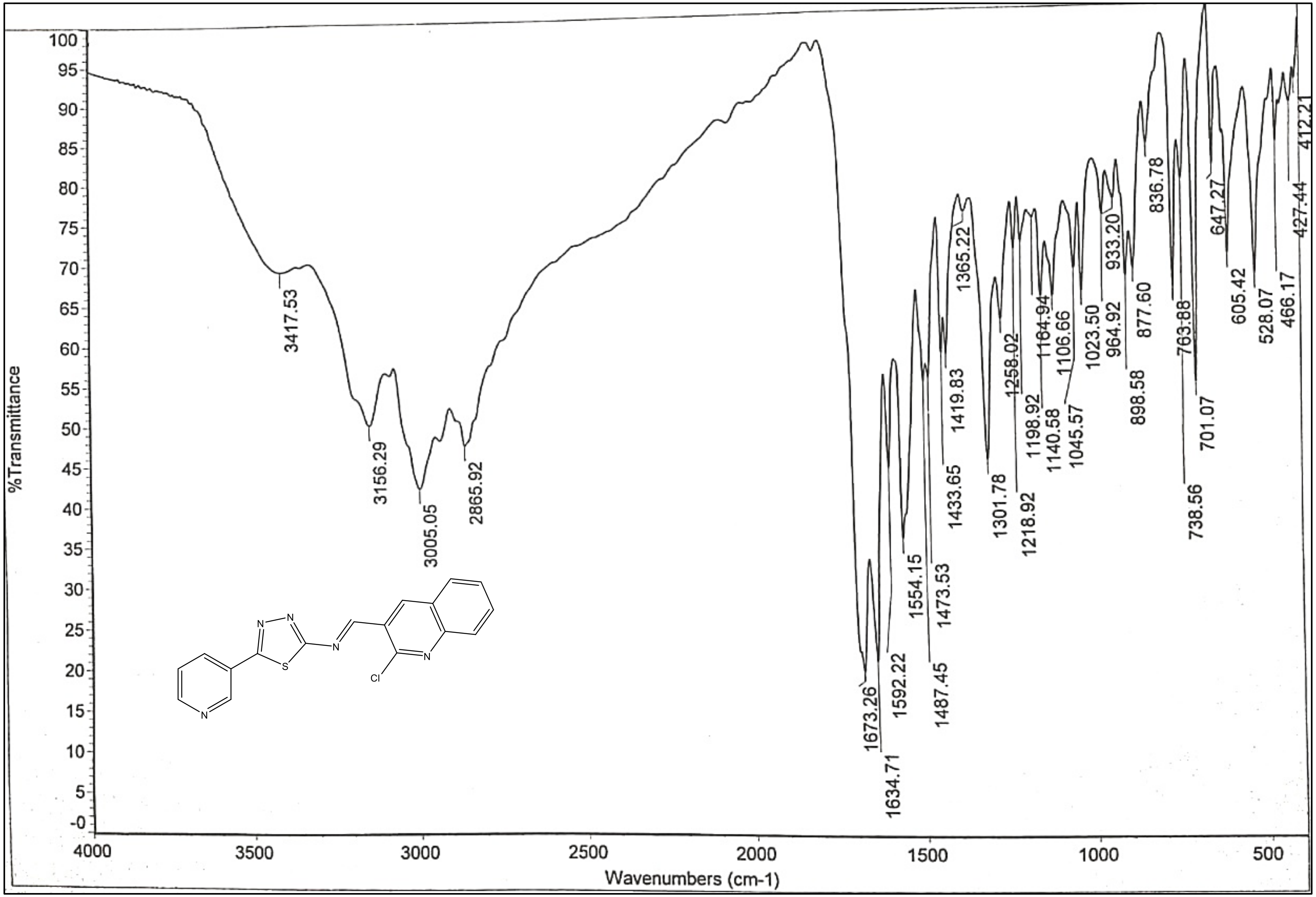
%Transmittance
Wavenumbers (cm-1)
3417.53
3156.29
3005.05
2865.92
1673.26
1634.71
1592.22
1554.15
1487.45
1473.53
1433.65
1419.83
1365.22
1301.78
1258.02
1218.92
1198.92
1164.94
1140.58
1106.66
1045.57
1023.50
964.92
933.20
898.58
877.60
836.78
763.88
738.56
701.07
647.27
605.42
528.07
466.17
427.44
412.21
N
S
Cl

IR spectrum *of 1-(2-chloroquinolin-3-yl)-N-(5-(pyridin-3-yl)-1,3,4-thiadiazol-2-yl)methanimine* **12**

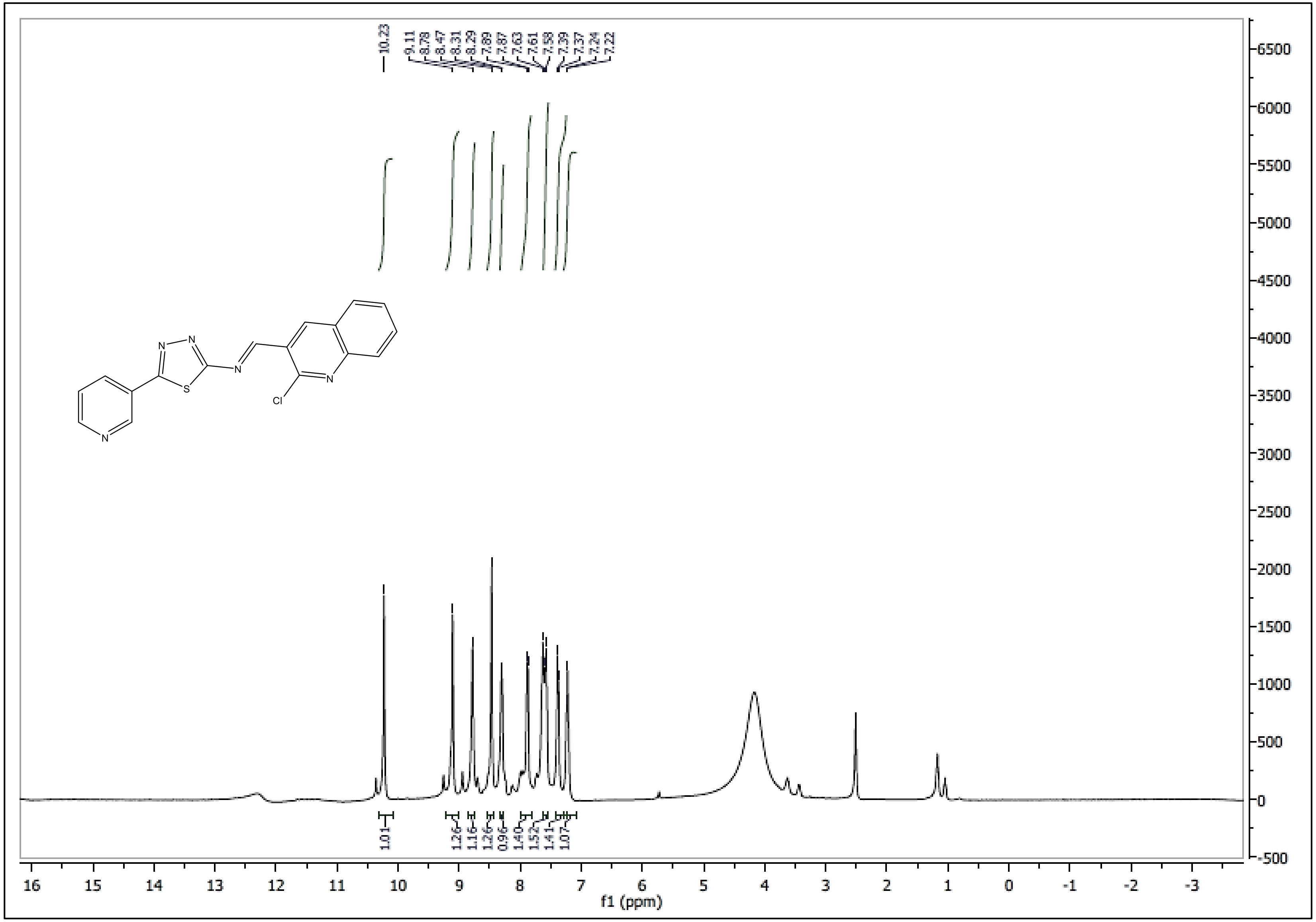

^{1}HNMR spectrum *of 1-(2-chloroquinolin-3-yl)-N-(5-(pyridin-3-yl)-1,3,4-thiadiazol-2-yl)methanimine* **12**

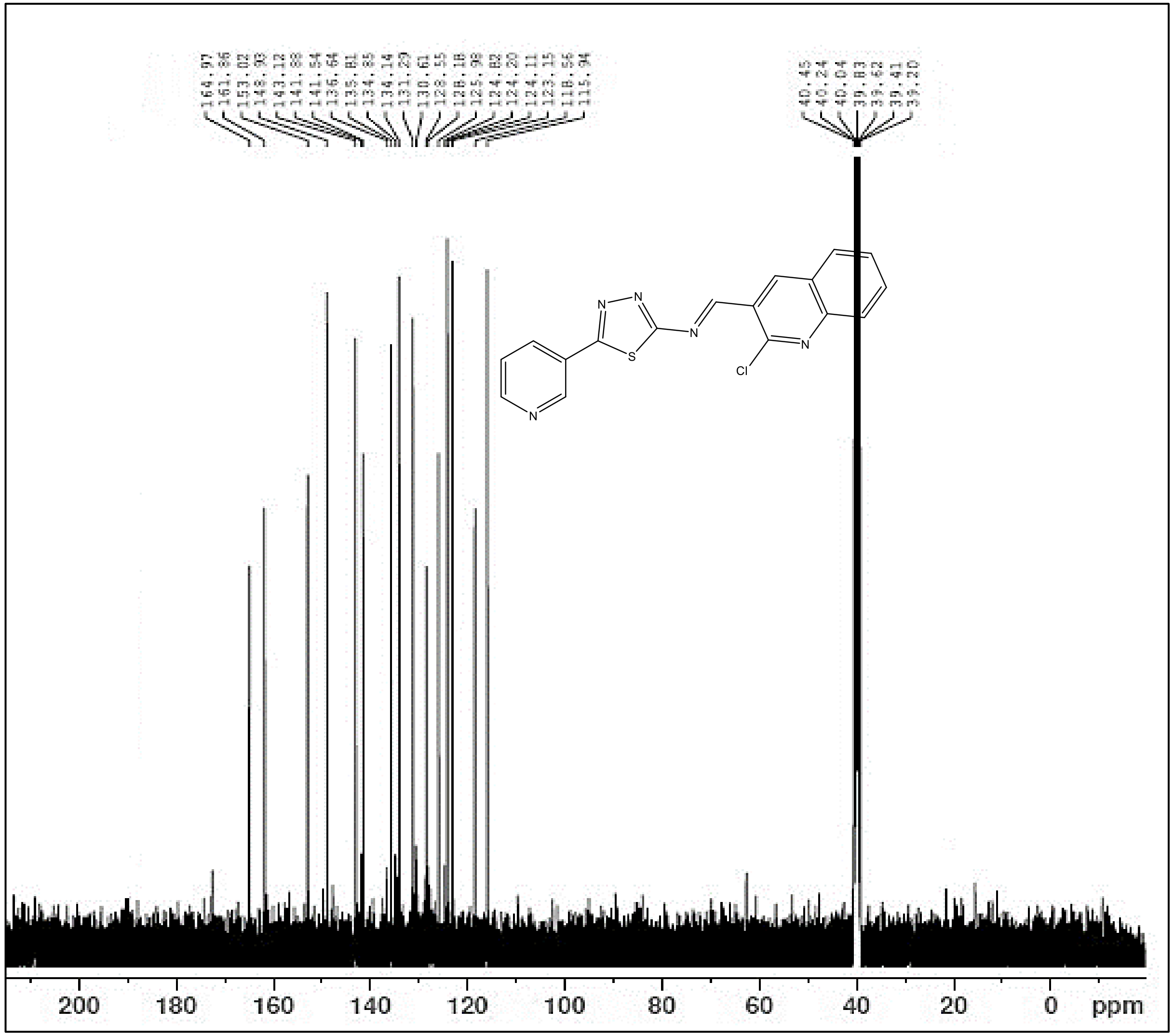

^{13}CNMR spectrum *of 1-(2-chloroquinolin-3-yl)-N-(5-(pyridin-3-yl)-1,3,4-thiadiazol-2-yl)methanimine* **12**

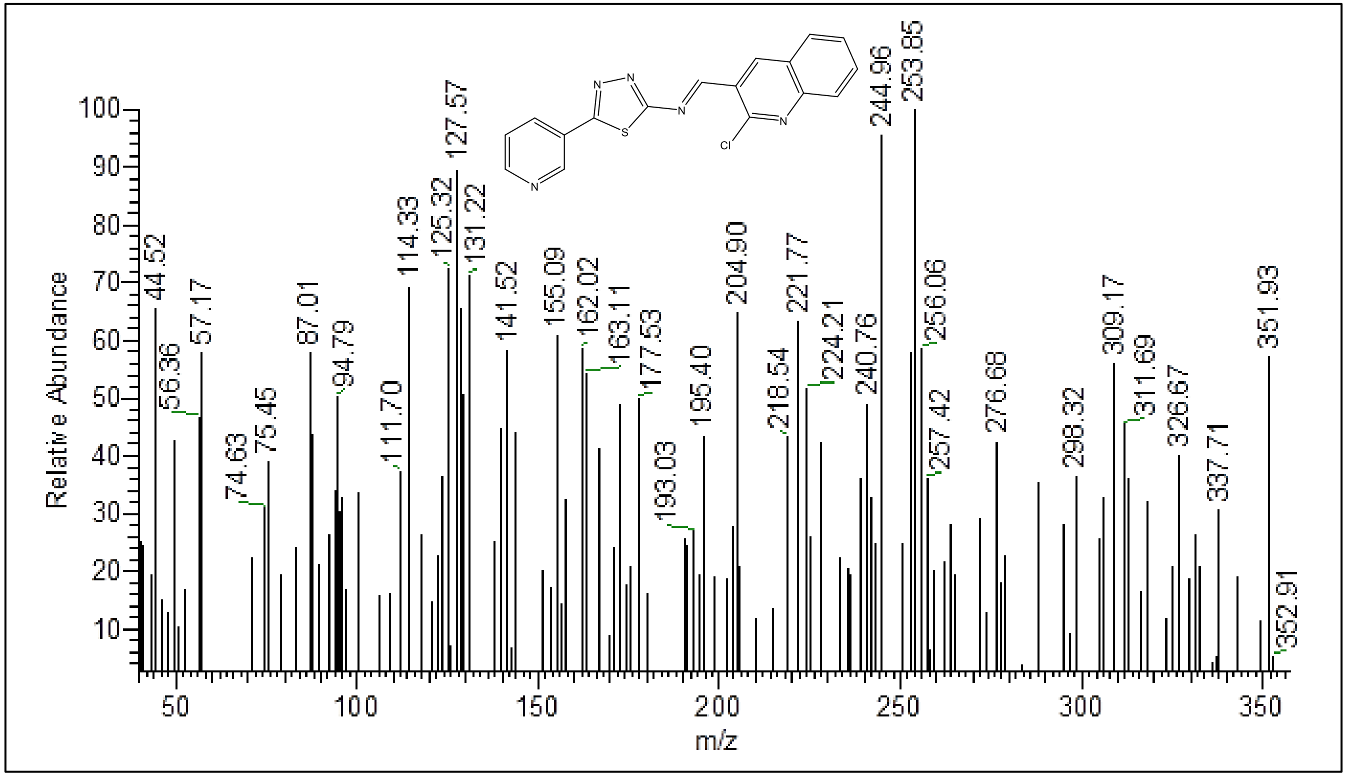

Mass spectrum *of 1-(2-chloroquinolin-3-yl)-N-(5-(pyridin-3-yl)-1,3,4-thiadiazol-2-yl)methanimine* **12**
